# VPS29 Exerts Opposing Effects on Endocytic Viral Entry

**DOI:** 10.1128/mbio.03002-21

**Published:** 2022-03-01

**Authors:** Daniel Poston, Yiska Weisblum, Alvaro Hobbs, Paul D. Bieniasz

**Affiliations:** a Laboratory of Retrovirology, The Rockefeller University, New York, New York, USA; b Weill Cornell/Rockefeller/Sloan-Kettering Tri-Institutional MD-PhD Program, New York, New York, USA; c Howard Hughes Medical Institute, The Rockefeller University, New York, New York, USA; Boston University School of Medicine

**Keywords:** SARS-CoV-2, COVID-19, coronavirus, influenza virus, ebolavirus, genome-wide CRISPR screen, respiratory viruses, VPS29, retromer, viral entry, trafficking, zoonosis, Ebola, endosomes, influenza, virus entry

## Abstract

Emerging zoonotic viral pathogens threaten global health, and there is an urgent need to discover host and viral determinants influencing infection. We performed a loss-of-function genome-wide CRISPR screen in a human lung cell line using HCoV-OC43, a human betacoronavirus. One candidate gene, VPS29, a component of the retromer complex, was required for infection by HCoV-OC43, SARS-CoV-2, other endemic- and pandemic-threat coronaviruses, as well as ebolavirus. Notably, we observed a heightened requirement for VPS29 by the recently described Omicron variant of SARS-CoV-2 compared to the ancestral variant. However, VPS29 deficiency had no effect on certain other viruses that enter cells via endosomes and had an opposing, enhancing effect on influenza A virus infection. Deficiency in VPS29 or other retromer components caused changes in endosome morphology and acidity and attenuated the activity of endosomal proteases. These changes in endosome properties caused incoming coronavirus, but not influenza virus particles, to become entrapped therein. Overall, these data show how host regulation of endosome characteristics can influence cellular susceptibility to viral infection and identify a host pathway that could serve as a pharmaceutical target for intervention in zoonotic viral diseases.

## INTRODUCTION

Because viruses rely on host cellular proteins to replicate, an attractive strategy for the next generation of antiviral therapies is targeted inhibition of human proteins—termed “dependency factors”—that are required for viral replication. Of particular interest are human proteins required by diverse viral lineages, encompassing not only known human pathogens but animal viruses that are of concern for future spillover into human populations. One universal aspect of the viral life cycle that could be targeted pharmacologically is viral entry. All enveloped viruses require fusion between viral and host cellular membranes for infection (1). Some enveloped viruses preferentially fuse at the plasma membrane, while others enter cells via endocytosis and fuse in compartments of the endolysosomal system (2). Viruses that fuse at the plasma membrane sometimes depend on the expression of cell surface proteases to activate viral fusion proteins, while viruses that enter through endosomes can be highly dependent on endosomal characteristics such as the presence of certain endosomal proteases and/or endosomal pH (3, 4).

The specific route of entry can dictate which dependency factors are required for productive infection. For example, the hemagglutinin (HA) of most influenza A virus (IAV) strains must be cleaved by trypsin-like proteases, which prime it for receptor binding and subsequent fusion (5). Like IAV, the spike protein of coronaviruses must also be processed by proteases in order to enter target cells. However, unlike HA, the spike protein often has two distinct cleavage sites, termed S1/S2 and S2′, that are cleaved at different stages of the virus replication cycle, specifically during biosynthesis (by the Golgi resident furin-like proteases) and during entry (by cell surface TMPRSS2 protease or endosomal cathepsins) (6). Cleavage regulates the liberation of the fusion peptide to enable fusion of the viral envelope with the cellular membranes, allowing infection to proceed. Similarly, the envelope protein of filoviruses, GP, requires two distinct cleavage steps. First, furin-mediated cleavage during exocytosis yields two subunits, GP1 and GP2, which remain linked by disulfide bonds to form the heterodimers that compose the trimeric envelope complex. Following endocytosis, GP1 is further cleaved by endosomal proteases, mainly cathepsins, in a process that removes the cap and the mucin-like domain to enable binding of GP1 to its endosomal receptor, Niemann-Pick C1 (NPC1) (7).

In this century alone, four emerging zoonotic respiratory pathogens—SARS-coronavirus (CoV), MERS-CoV, H1N1 influenza A virus (IAV), and SARS-CoV-2—have caused significant morbidity and mortality. Of these, SARS-CoV, MERS-CoV, and SARS-CoV-2 are all enveloped, positive-stranded RNA viruses in the genus betacoronavirus (8). Four other coronaviruses are known to infect humans; human CoV (HCoV)-OC43 and HCoV-HKU1 are members of the betacoronavirus genus (9), while HCoV-229E and HCoV-NL63 are members of the alphacoronavirus genus. Each generally causes only mild illness. To identify coronavirus dependency factors, we performed a genome-wide loss-of-function CRISPR screen using HCoV-OC43 in a human lung cell line and focused on candidate hits that are required by diverse coronaviruses. We identified one such factor, VPS29, that is broadly required by both human and animal CoVs. VPS29 is a component of both retromer (VPS26/VPS29/VPS35) and retriever (DSCR3/VPS29/C16orf62) complexes, two distinct but related complexes that, together with the CCDC22/CCDC93/COMMD (CCC) complex, mediate endosome-to-plasma membrane and endosome-to-*trans*-Golgi network (TGN) recycling of transmembrane cargo (10–13). We show that loss of VPS29 impairs CoV infection and also causes failure of ebolavirus infection. In stark contrast, we show that VPS29 deficiency facilitates IAV infection. We further show that VPS29 deficiency causes profound changes in endosomal properties, including alteration of morphology, acidity, and proteolytic activity that differentially impact the egress of viral genetic material from endosomes.

## RESULTS

### A genome-wide screen reveals HCoV-OC43 dependency factors.

**FIG 1:**
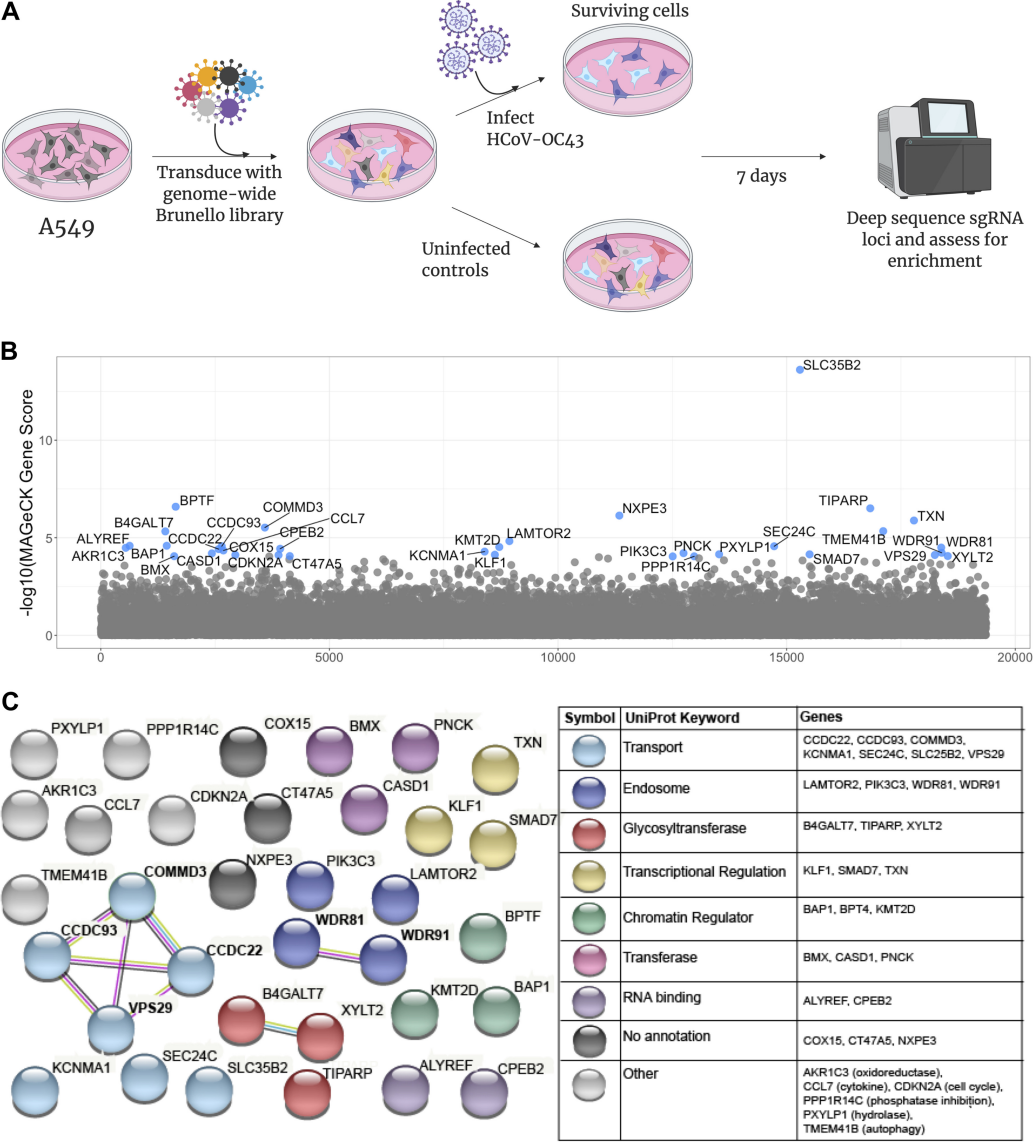
A CRISPR screen reveals genes influencing HCoV-OC43 susceptibility. (A) Schematic of screening setup. (B) Screen results, where the *x* axis corresponds to each unique gene in the library (labeled randomly from 1 to 19,114), and the *y* axis denotes the log_10_ MAGeCK gene score. All genes scoring higher than the best-scoring nontargeting control pseudogene are labeled in blue. The screen was performed in three independent replicates. (C) STRING database analysis and UniProt annotation of gene hits. Sphere colors correspond to UniProt keywords, and connecting lines indicate the strength of evidence underlying gene-gene interactions (pink, experimentally determined interaction; blue, annotated interaction in curated databases; gray, evidence of coexpression; yellow, text mining).

To identify host proteins required for HCoV-OC43 infection, we performed a genome-wide CRISPR screen in the A549 lung adenocarcinoma cell line. Briefly, A549 cells were transduced with the Brunello single guide RNA (sgRNA) library (14, 15) at a low multiplicity of infection (MOI) (0.3) and high coverage (500×) to generate a population of cells, each harboring a single sgRNA. After selection to remove untransduced cells, A549-Brunello cells were infected with HCoV-OC43 at an MOI of 0.1 and incubated for 1 week to allow viral-induced cell death to occur (Fig. 1A). Enrichment of sgRNA sequences in the surviving cells—i.e., those putatively lacking a dependency factor—was assessed using MAGeCK (16).

We identified 34 candidate dependency factors, defined as genes scoring higher than the highest-scored nontargeting control (Fig. 1B). As a positive control, we identified CASD1, the enzyme responsible for the generation of 9-*O*-acetylated sialic acids, which serve as the receptor for HCoV-OC43 (17). Consistent with several other genome-wide screens for viral dependency factors, we identified multiple genes (SLC35B2, XYLT2, and B4GALT7) involved in heparan sulfate biosynthesis, implying that heparan sulfate is an attachment factor for HCoV-OC43 ([Bibr B18][Bibr B19] 22).

To further classify gene hits (Fig. 1C), we performed a functional enrichment analysis using the STRING database followed by annotation with UniProt keywords (23, 24). Many of the hits were associated with intracellular transport or endosome activity, including VPS29, the **C**CDC22/**C**CDC93/**C**OMMD3 (CCC) complex, and the WDR81/91 complex, suggesting a requirement for these functions in HCoV-OC43 infection. Additionally, we identified PIK3C3, which generates phosphatidylinositol 3-phosphate [PI(3)P], a phospholipid required for the recruitment of retromer to endosomes (25). Some of the genes identified by our screen were also recently reported in CRISPR screens utilizing SARS-CoV-2, implying that they are broadly required for coronavirus infection (26, 27).

### The requirement for candidate host factors is both cell type and virus dependent.

We next investigated whether the VPS29/CCC complex and the WDR81/91 were required for infection by a diverse panel of respiratory viruses, including coronaviruses. In addition to HCoV-OC43, we tested additional seasonal HCoVs (HCoV-NL63 and HCoV-229E), recombinant vesicular stomatitis virus (rVSV)/SARS-CoV-2, a chimeric vesicular stomatitis virus encoding the SARS-CoV-2 spike protein, as well as other pathogenic respiratory viruses, including IAV, adenovirus, and respiratory syncytial virus (RSV). We used CRISPR/Cas9 to generate individual cell lines lacking each gene of interest and confirmed knockout (KO), both by sequencing target loci and by Western blot analyses (Fig. S1A in the supplemental material). Importantly, KO of these genes did not affect cellular viability or proliferation. Because viral dependency factors identified via CRISPR screening might be required in a cell type-specific manner, we evaluated the requirement of these genes for infection in multiple cell lines expressing ACE2 (the receptor for both SARS-CoV-2 and HCoV-NL63), specifically A549-ACE2, HT1080-ACE2, and 293T-ACE2.

10.1128/mBio.03002-21.1 FIG S1 Validation of CRISPR KO and VPS29 reconstitution. (A) Western blotting confirmation of VPS29, CCDC22, CCDC93, and COMMD3 KO. (B) Western blotting confirmation of VPS29 SCC KO and reconstitution. Antibodies used are VPS29 (Abcam; catalog no. ab236796), CCDC22 (Protein Tech; catalog no. 16636-1-AP), CCDC93 (Protein Tech; catalog no. 20861-1-AP), and COMMD3 (Protein Tech; catalog no. 26240-1-AP). (C) Quantification of the level of VPS29 expression normalized to GAPDH (glyceraldehyde-3-phosphate dehydrogenase). Download FIG S1, PDF file, 0.2 MB. Copyright © 2022 Poston et al. 2022 Poston et al. https://creativecommons.org/licenses/by/4.0/ This content is distributed under the terms of the Creative Commons Attribution 4.0 International license.

**FIG 2:**
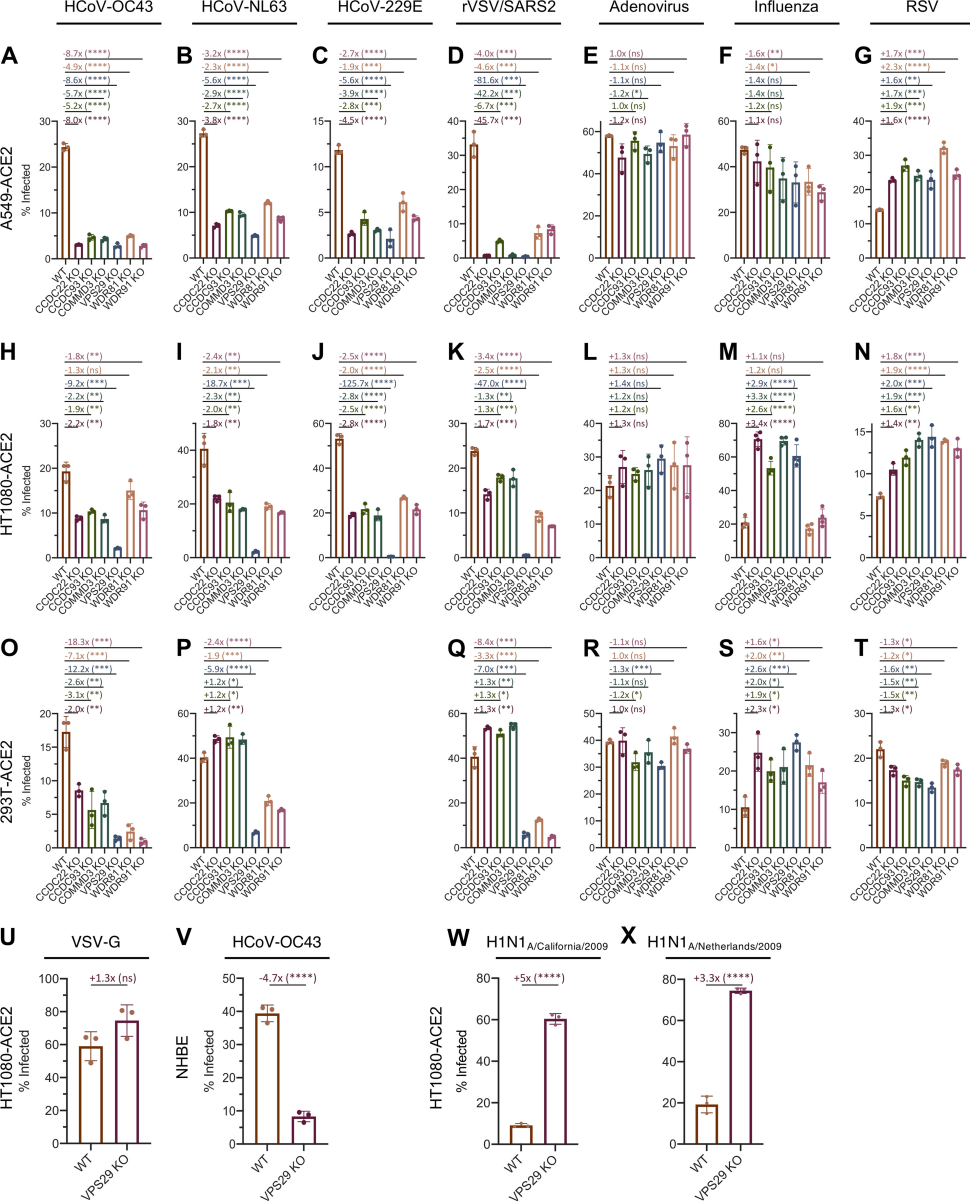
Requirement for identified host proteins is cell type and virus dependent. (A to X) Cells were infected with the indicated viruses at an MOI of 0.3. At 24 h postinfection, cells were stained, and the percentage of infected cells was determined by flow cytometry. (A to G) A549-ACE2; (H to N, U, and W to X) HT1080-ACE2; (O to T) 293T-ACE2; (V) NHBE. Mean (bar graph) of three replicates (dots). Error bars indicate SD. Data shown are representative of at least two independent experiments. Statistical test, Student's *t* test between WT and KO cells. *, *P < *0.05; **, *P < *0.01; ***, *P < *0.001; ****, *P < *0.0001; ns, not significant.

Given their function in endosomal trafficking, we hypothesized that these hits would most likely affect viral entry. We therefore performed short-term infection assays and quantified infected cells via flow cytometry. There was a strong requirement for VPS29/CCC complex as well as WDR81/91 in A549 cells for all CoVs tested (Fig. 2A to D). However, the was no requirement of these factors for IAV, adenovirus, or RSV infection of A549 cells (Fig. 2E to G). In all other cell lines tested, there was a strong requirement for VPS29 for all coronaviruses, but minimal dependency on VPS29 or the other candidate proteins was found for adenovirus and RSV (Fig. 2H to T). Since these viruses all rely on endocytic pathways for viral entry (28–30), these data indicate that VPS29/CCC and WDR81/91 are specifically required for coronavirus infection, rather than broadly impairing endocytic function. The magnitude of the effect of CCC complex and WDR81/91 knockout on CoV infection was different in different cell lines. For example, KO of the CCC complex or WDR81/91 had a blunted effect on CoV infection in HT1080-ACE2 cells (Fig. 2H to K). Moreover, in 293T-ACE2 cells, KO of the CCC complex inhibited HCoV-OC43 but not HCoV-NL63 or rVSV/SARS-CoV-2 infection, while WDR81/91 knockout impaired infection for all three viruses (Fig. 2O to Q).

We found that VSV infection was unaffected by VPS29 KO (Fig. 2U). Because the sole difference between rVSV/SARS-CoV-2 and VSV itself is that rVSV/SARS-CoV-2 enters cells using the SARS-CoV-2 spike protein in lieu of VSV glycoprotein (VSV-G), these data suggest that it is the entry pathway that imposes the requirement for VPS29. Given the strong requirement for VPS29 by all tested HCoVs, in all cell lines tested, we sought to further confirm the relevance of VPS29 to HCoV infection. To do so, we used CRISPR/Cas9 to knock out VPS29 in normal human bronchial epithelial (NHBE) primary lung cells. Loss of VPS29 strongly inhibited HCoV-OC43 infection in NHBE cells (Fig. 2V), suggesting that VPS29 is important for HCoV infection of physiologically relevant cells.

In contrast to effects on coronavirus infection, we observed precisely the opposite effect of VPS29 or CCC complex deficiency on IAV infection in HT1080-ACE2 and 293T-ACE2 cells. That is, KO of VPS29 or CCC complex components enhanced IAV infection (Fig. 2M and S), while WDR81/91 KO had no effect. To confirm the phenotype observed using the IAV strain A/WSN/33, we analyzed two separate strains of 2009 pandemic H1N1 IAV, A/Netherlands/602/2009 (H1N1)pdm09 (H1N1_2009 Netherlands_), and A/California/04/2009 (H1N1)pdm09 (H1N1_2009 California_). We found that the ability of VPS29 KO to enhance IAV entry was conserved in the pandemic IAV strains (Fig. 2W and X). That the same set of endocytic factors could promote infection of coronaviruses while antagonizing IAV infection indicates endosome-based viral entry pathways are influenced by specific sets of host proteins that can facilitate or restrict viral entry.

### VPS29-associated proteins facilitate CoV infection and hinder IAV infection.

Because of the opposing effects of VPS29 on HCoV and IAV infection, we elected to examine this protein in more detail, specifically in HT1080 cells, where VPS29 KO strongly suppressed CoV infection and facilitated IAV infection. VPS29 can participate in multiple different protein complexes with distinct roles in normal cell biology (10). Thus, in order to clarify which VPS29-interacting proteins, if any, are important for facilitating CoV infection and inhibiting IAV infection, we performed a focused small interfering RNA (siRNA) screen targeting VPS29-interacting proteins and assessed the impact of knockdown (KD) on HCoV and IAV infection.

**FIG 3:**
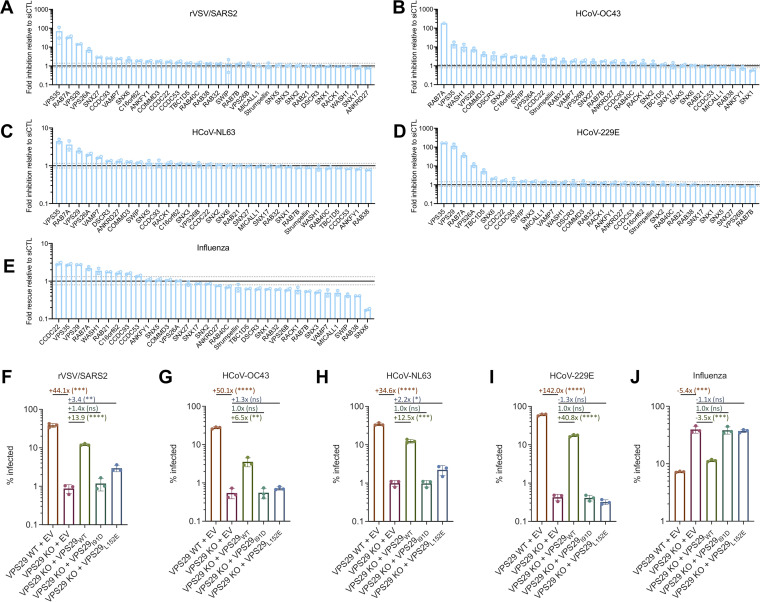
Effect of VPS29 KO on HCoV and IAV infection is primarily driven by loss of retromer/WASH complex function. (A to E) HT1080 cells were transfected with a focused siRNA library targeting VPS29-interacting proteins. Two days after transfection, cells were infected with rVSV/SARS-CoV-2 (A), HCoV-OC43 (B), HCoV-NL63 (C), HCoV-229E (D), and IAV (E) at an MOI of 0.3. At 24 h postinfection, cells were stained, and the percentage of infected cells was determined by flow cytometry. Plotted are levels of inhibition (for HCoVs and rVSV/SARS-CoV-2) or increase (for IAV) in siRNA KD cells relative to siRNA nontargeting control. Fold change values were calculated by comparing levels of infection in KD cells to the average of 4 separate pools of nontargeting siRNA controls. Solid black line marks fold change of 1. The dashed lines mark the highest and lowest fold changes of nontargeting siRNA controls from the average. (F to J) WT and VPS29 mutants were reconstituted in VPS29 KO cells. Cells were infected with rVSV/SARS-CoV-2 (F), HCoV-OC43 (G), HCoV-NL63 (H), HCoV-229E (I), or IAV (J). At 24 h postinfection, cells were stained, and the percentage infected cells was determined by flow cytometry. Mean (bar graph) of three replicates (dots). Error bars indicate SD. Data shown are representative of two independent experiments. Statistical test, Student's *t* test between VPS29 KO cells and VPS29-expressing cells. *, *P < *0.05; **, *P < *0.01; ***, *P < *0.001; ****, *P < *0.0001; ns, not significant.

Knockdown of VPS26A, VPS29, VPS35, or RAB7A each impaired HCoV-OC43, HCoV-NL63, HCoV-229E, and rVSV/SARS-CoV-2 infection (Fig. 3A to D). These data strongly suggest that the participation of VPS29 in the retromer complex (VPS26A/VPS29/VPS35), which is recruited to endosomes via Rab7A, is the means by which it facilitates CoV infection (31). Interestingly, KD of DSCR3 and C16orf62, which play analogous roles to VPS26 and VPS35 and form the retriever complex (11), inhibited HCoV-OC43 infection but not HCoV-NL63, HCoV-229E, or rVSV/SARS-CoV-2 infection.

IAV infection was enhanced by KD of an overlapping set of VPS29-associated proteins, specifically CCDC22, VPS35, VPS29, RAB7A, WASH1, and RAB21 (Fig. 3E). WASH1 is a member of the WASH complex, which facilitates formation of actin patches on endosomes and interacts with and is critical for some protein-sorting functions of retromer (32). RAB21 is a known effector of the WASH complex (33). These data thus suggest that the enhancement of IAV infection in VPS29 KO cells is due to the absence of an intact retromer/WASH complex. While KO or KD of VPS29 facilitates IAV infection, KD of some VPS29-interacting proteins impaired IAV infection. For example, KD of SNX6 impaired IAV infection >5-fold. However, KD of SNX6 did not affect HCoV infection, indicating that the inhibition of IAV infection is not simply due to global impairment of endosomal function due to SNX6 KD.

### The ability of VPS29 to facilitate CoV infection and inhibit IAV infection depends on interaction with retromer components and regulators.

In an orthogonal approach to investigate the role of the retromer complex in facilitating CoV infection and hindering IAV infection, we generated HT1080 VPS29 KO single-cell clones (SCCs) and reconstituted them with wild-type (WT) and mutant forms of VPS29. One VPS29 mutant (I91D) does not interact with the retromer component VPS35, while the other (L152E) does not interact with TBC1D5, a RAB7A GTPase-activating protein that is critical for endosomal recycling of known retromer cargoes (34–36). In agreement with our previous data, CoV infection was inhibited and IAV infection was enhanced in the VPS29 KO SCC (Fig. 3F to J). Susceptibility of HT1080 VPS29 KO cells was substantially restored upon reconstitution with a construct expressing a WT sgRNA-resistant VPS29 (Fig. S1B and C). However, reconstitution with a construct expressing VPS29_I91D_ or VPS29_L152E_ did not reverse the effects of VPS29 KO on HCoV or IAV infection (Fig. 3F to J). Overall, these data confirm that loss of the retromer complex function is the major means by which VPS29 KO affects CoV and IAV infection.

### VPS29 deficiency results in enlarged, deacidified endosomes.

To elucidate the impact of VPS29 on viral infection, we next investigated the impact of VPS29 KO on normal endosomal function. We labeled endosomes in living cells using a construct containing two FYVE domains fused to Scarlet (2×FYVE-mSCAR), which binds to PI(3)P that is enriched on endosome membranes (37). Thereafter, we treated cells with dextran labeled with pH-sensitive (pHrodo Green or pHrodo Red) or pH-insensitive (Alexa Fluor [AF] 488) fluorophores to visualize endocytic cargo uptake, as well as the pH status of these endosomes.

**FIG 4:**
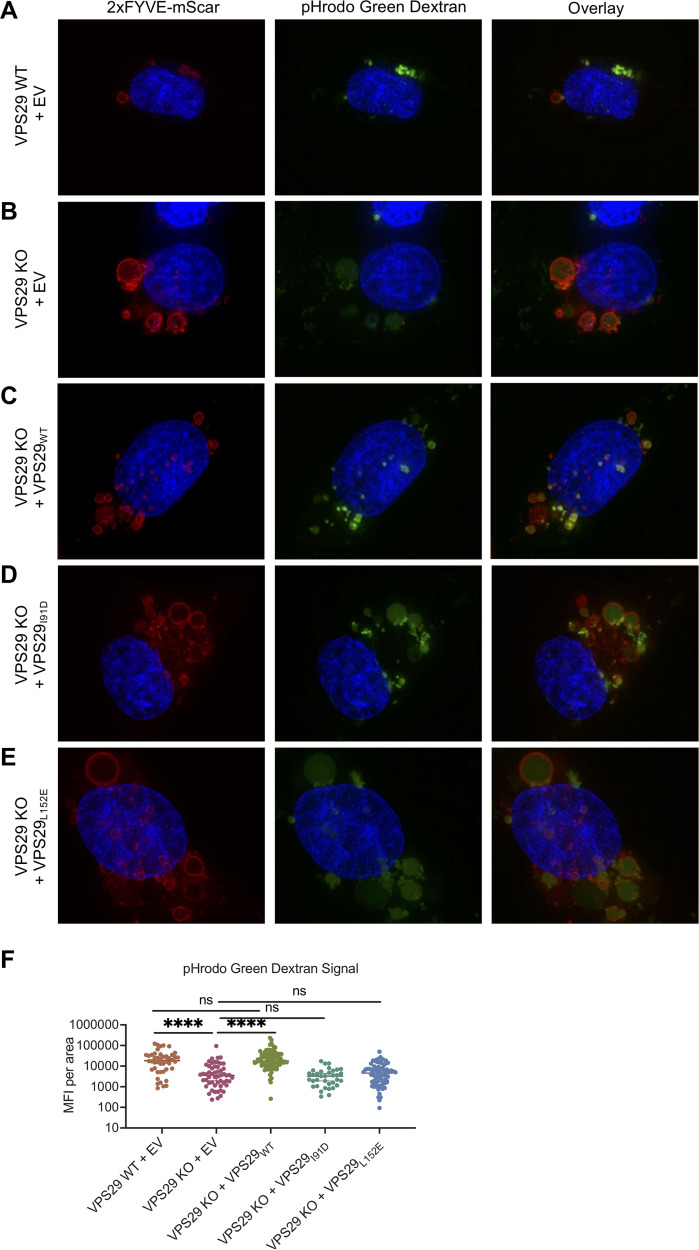
VPS29-KO results in enlarged, deacidified PI(3)P-rich vesicles. Representative images of HT1080 cells transduced with a construct expressing 2×FYVE-mSCAR after incubation with pHrodo Green dextran for 60 min. (A) VPS29 WT plus EV expression cassette. (B) VPS29 KO HT1080 plus EV expression cassette. (C) VPS29 KO HT1080 reconstituted with WT VPS29. (D) VPS29 KO HT1080 reconstituted with VPS29_I91D_. (E) VPS29 KO HT1080 reconstituted with VPS29_L152E_. EV, empty vector. (F) Quantification of mean fluorescence intensity (MFI) of pHrodo Green dextran signal inside 2×-FYVE-labeled endosomes from *n *= 4 independent images (images in panels A to E, as well as the additional representative images depicted in Fig. S2 in the supplemental material). Error bars indicate SD. Statistical test used is Student's *t* test. *, *P < *0.05; **, *P < *0.01; ***, *P < *0.001; ****, *P < *0.0001; ns, not significant.

**FIG 5:**
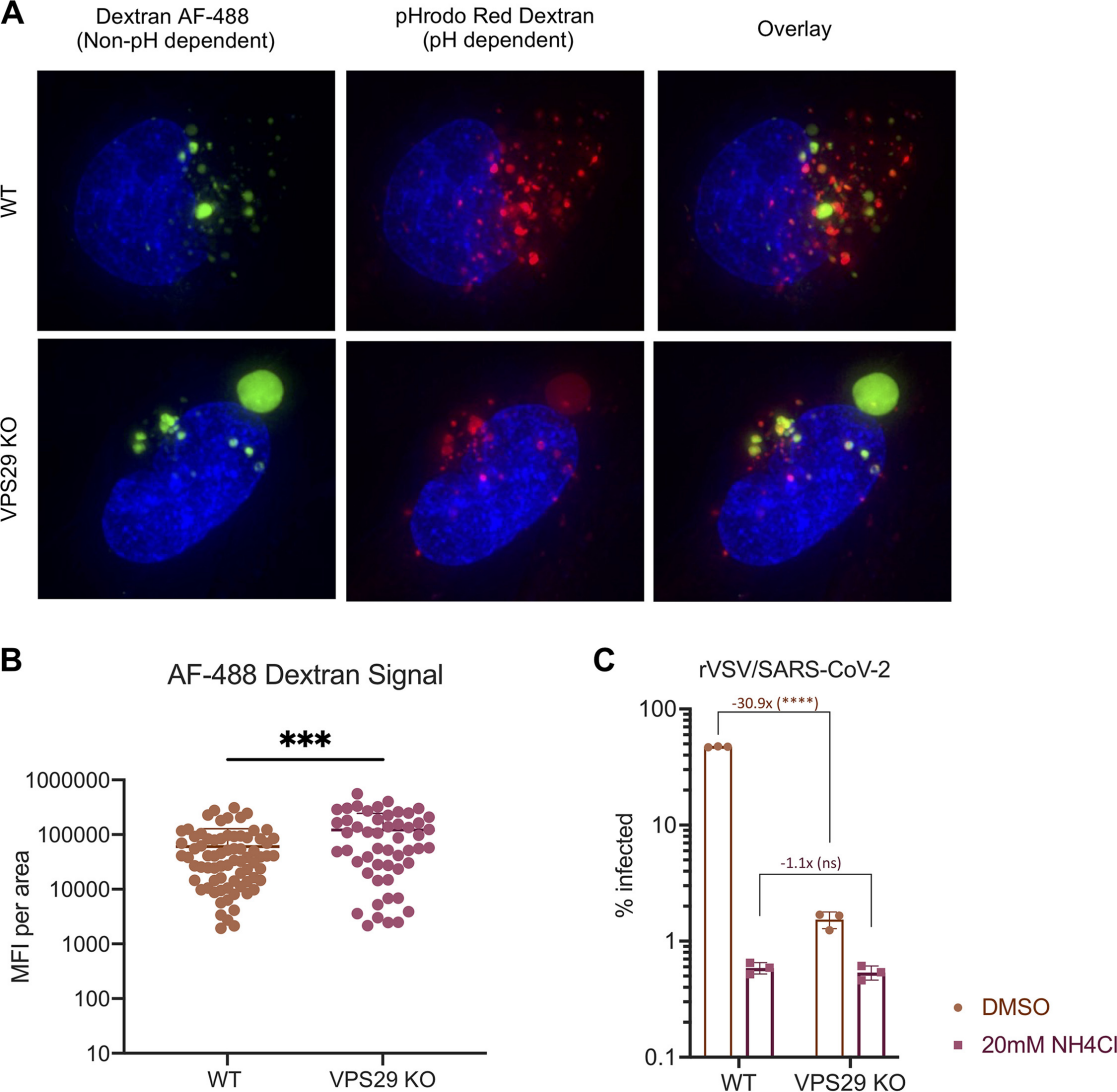
Enlarged, deacidified vesicles in VPS29-KO cells are not impaired for cargo loading. (A) Representative images of HT1080 cells incubated with dextran AF-488 (non-pH dependent) and pHrodo Red dextran (pH dependent) for 60 min. (B) Quantification of mean fluorescence intensity (MFI) of AF-488 dextran signal inside vesicles in WT and VPS29 KO cells from *n *= 3 independent images (images in panel A, as well as the additional representative images in Fig. S3 in the supplemental material). (C) WT and VPS29 KO HT1080 cells were treated with 20 mM NH_4_Cl for 60 min before infection with rVSV/SARS-CoV-2. At 24 h postinfection, the percentage of infected cells was determined by flow cytometry. Error bars indicate SD. Statistical test used was Student's *t* test between WT and KO cells. *, *P < *0.05; **, *P < *0.01; ***, *P < *0.001; ****, *P < *0.0001; ns, not significant.

Unlike parental HT1080 cells, VPS29 KO cells displayed a prominent subset of enlarged PI(3)P-positive endosomes. These enlarged endosomes were deacidified, as evident from decreased pHrodo Green dextran signal compared to endosomes in unmanipulated cells or other smaller endosomes in VPS29 KO cells (Fig. 4A and B and Fig. S2A). Importantly, there was a return to normal endosome phenotype after reconstitution with wild-type VPS29, confirming that this effect is due to VPS29 KO (Fig. 4C and Fig. S2). The appearance of enlarged, deacidified vesicles was maintained in VPS29 KO cells reconstituted with VPS29_I91D_ or VPS29_L152E_ (Fig. 4D and E and Fig. S2), suggesting that this phenotype is due to retromer disfunction. To confirm that this phenotype is due to retromer disfunction, we performed similar experiments in cells treated with siRNA duplexes targeting distinct retromer components, namely, VPS26A or VPS35. We found that, as in VPS29 knockdown cells, loss of VPS26A or VPS35 also resulted in enlarged, deacidified vesicles, providing further evidence that the effect seen in VPS29 KO cells is due to loss of retromer (Fig. S2B). Interestingly, KO of the CCC complex, but not WDR81/91, also resulted in a similar phenotype (Fig. S2C). Quantification of the pH-sensitive dextran signal from these images of VPS29 KO cells revealed a 3.7-fold decrease in fluorescence intensity (Fig. 4F) that is rescued upon reconstitution with WT VPS29, but not with VPS29_I91D_ or VPS29_L152E._ Importantly, the enlarged endosomes in VPS29 KO cells exhibited equivalent fluorescent intensity to endosomes in normal cells when cells were incubated with pH-insensitive AF-488 dextran, indicating that while they were deacidified, they were not impaired in cargo loading (Fig. 5A and B and Fig. S3).

10.1128/mBio.03002-21.2 FIG S2 Additional representative images demonstrating VPS29 KO results in enlarged, deacidified PI(3)P-rich vesicles and quantification showing that loss of other members of the retromer/CCC complex also results in deacidified vesicles. (A) Additional representative images from Fig. 4. HT1080 cells transduced with 2×FYVE-mSCAR after incubation with pHrodo Green dextran for 60 minutes. (B and C) Quantification of mean fluorescence intensity (MFI) of pHrodo Green dextran signal inside 2×FYVE-labeled endosomes. (B) HT1080 transfected with siRNA-targeting retromer proteins VPS29, VPS26A, and VPS35. (C) HT1080 WT or KO for the indicated gene. MFI was quantified from *n *= 3 independent images. Statistical test used is Student’s *t* test. *, *P < *0.05; **, *P < *0.01; ***, *P < *0.001; ****, *P < *0.0001. Download FIG S2, PDF file, 0.7 MB. Copyright © 2022 Poston et al. 2022 Poston et al. https://creativecommons.org/licenses/by/4.0/ This content is distributed under the terms of the Creative Commons Attribution 4.0 International license.

10.1128/mBio.03002-21.3 FIG S3 Additional representative images showing the enlarged, deacidified vesicles in VPS29 KO cells are not impaired for cargo loading. Additional representative images from Fig. 5. WT and VPS29 KO HT1080 cells incubated for 60 minutes with an equal molar ratio of pHrodo dextran Red of 10,000 MW and dextran AF-488 of 10,000 MW. Download FIG S3, PDF file, 0.1 MB. Copyright © 2022 Poston et al. 2022 Poston et al. https://creativecommons.org/licenses/by/4.0/ This content is distributed under the terms of the Creative Commons Attribution 4.0 International license.

Overall, these data suggest that impaired endosome acidification in VPS29 KO cells is responsible for the impairment of CoV infection. In order to test this, we artificially deacidified endosomes by treating WT or VPS29 KO cells with NH_4_Cl. As expected, there was a dramatic decrease in rVSV/SARS-CoV-2 infectivity in WT cells treated with NH_4_Cl (Fig. 5C). Interestingly, this effect was much less pronounced in VPS29 KO cells, and there was no further impairment in infectivity in VPS29 KO cells treated with NH_4_Cl than there was in WT cells treated with NH_4_Cl. That lack of additive effect of NH_4_Cl treatment in VPS29 KO cells suggests both conditions result in an analogous effect on viral infection.

### VPS29 KO results in entrapment of rVSV/SARS-CoV-2 in endosomes.

The above findings suggested that CoV infection is impaired in VPS29 KO cells due to impediment in spike-dependent egress from endosomes. To directly test this idea, we generated rVSV/SARS-CoV-2_NG-P_, a replication-competent chimeric VSV expressing SARS-CoV-2 spike protein in lieu of VSV-G, and containing the VSV structural protein P fused to mNeonGreen (NG-P), thus enabling the direct observation of entering viral particles (38).

**FIG 6:**
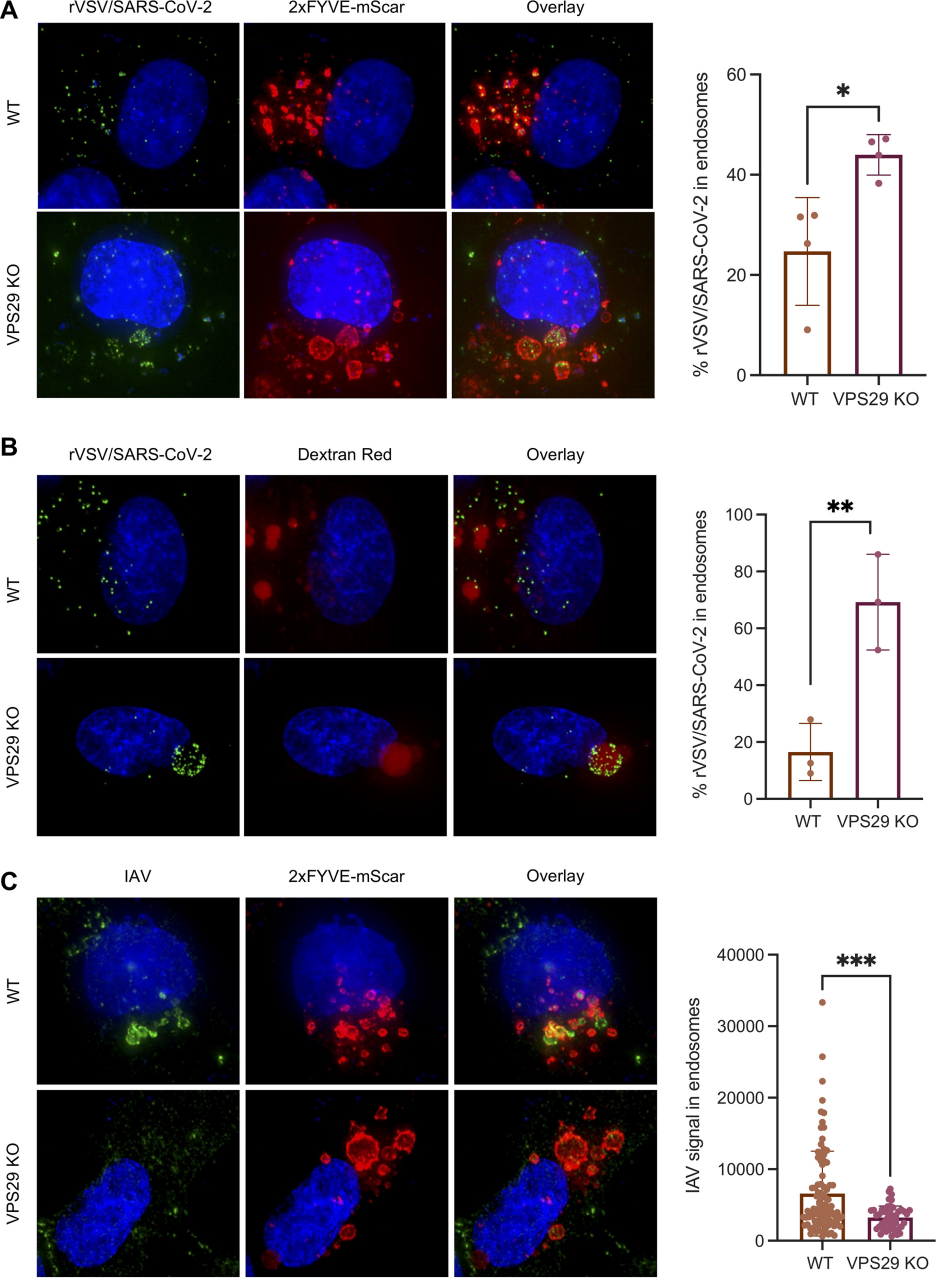
VPS29 KO results in rVSV/SARS-CoV-2 specifically remaining trapped in endosomes. (A) Representative images of rVSV/SARS-CoV-2_NG-P_ infection in WT and VPS29 KO HT1080 cells. 2×FYVE-mSCAR-labeled cells were infected with rVSV/SARS-CoV-2_NG-P_ for 60 min. Quantification indicates the percentage of rVSV/SARS-CoV-2_NG-P_ punctae inside 2×FYVE-labeled endosomes from *n *= 4 independent images (images in panel A, as well as the additional representative images in Fig. S4A in the supplemental material). (B) Representative images of WT and VPS29 KO HT1080 cells incubated for 60 min with dextran Red 10,000 MW and rVSV/SARS-CoV-2_NG-P._ Quantification indicates the percentage of rVSV/SARS-CoV-2_NG-P_ punctae inside dextran Red-labeled endosomes from *n *= 4 independent images (images in panel B, as well as the additional representative images in Fig. S4B). (C) Representative images of IAV infection in WT and VPS29 KO HT1080 cells labeled with 2×FYVE-mSCAR. Cells were infected with IAV for 60 min then fixed and stained for IAV NP. Quantification indicates the IAV signal (MFI) inside 2×FYVE-labeled endosomes from *n *= 4 independent images (images in panel C, as well as the additional representative images in Fig. S5). Error bars indicate SD. Statistical test used was Student's *t* test. *, *P < *0.05; **, *P < *0.01; ***, *P < *0.001.

At 60 min postinfection of parental HT1080 cells, few NG-P punctae were evident within 2×FYVE-mSCAR labeled endosomes, suggesting successful egress of most rVSV/SARS-CoV-2_NG-P_ particles (Fig. 6A and Fig. S4A) and minimal accumulation therein. However, in VPS29 KO cells, enlarged endosomes contained many rVSV/SARS-CoV-2_NG-P_ punctae at 60 min after infection. Likewise, when cells were infected in the presence of labeled dextran and imaged 60 min postinfection, we observed a similar phenotype with rVSV/SARS-CoV-2 particles accumulated in enlarged, dextran-containing vesicles in VPS29 KO cells (Fig. 6B and Fig. S4B). Overall, these data indicate that the major inhibitory effect of VPS29 KO on CoV infection is the result of failed egress from endosomes.

10.1128/mBio.03002-21.4 FIG S4 Additional representative images demonstrating VPS29 KO results in rVSV/SARS-CoV-2 remaining trapped in vesicles. (A) Additional representative images from Fig. 6A. rVSV/SARS-CoV-2_NG-P_ infection in WT and VPS29 KO HT1080 cells. Cells were infected with rVSV/SARS-CoV-2_NG-P_ for 60 minutes. (B) Additional representative images from Fig. 6B. WT and VPS29 KO HT1080 cells incubated for 60 minutes with dextran Red 10,000 MW and rVSV/SARS-CoV-2_NG-P_. Download FIG S4, PDF file, 0.5 MB. Copyright © 2022 Poston et al. 2022 Poston et al. https://creativecommons.org/licenses/by/4.0/ This content is distributed under the terms of the Creative Commons Attribution 4.0 International license.

10.1128/mBio.03002-21.5 FIG S5 Additional representative images demonstrating that IAV is more associated with PI(3)P-rich endosomal membranes in WT HT1080 cells than in VPS29 KO HT1080 cells. Additional representative images from Fig. 6C. IAV infection in WT and VPS29 KO HT1080 cells labeled with 2×FYVE-mSCAR. Cells were infected with IAV for 60 minutes then fixed and stained for IAV NP. Download FIG S5, PDF file, 0.3 MB. Copyright © 2022 Poston et al. 2022 Poston et al. https://creativecommons.org/licenses/by/4.0/ This content is distributed under the terms of the Creative Commons Attribution 4.0 International license.

Similar experiments in which incoming IAV virions were detected by immunofluorescence 60 min after (Fig. 6C and Fig. S5) revealed that IAV particles did not accumulate in the enlarged 2×FYVE-mSCAR-labeled endosomes in VPS29 KO cells. Thus, the effect of VPS29 KO on rVSV/SARS-CoV-2 was indeed specific. In fact, there was a significantly greater association between incoming IAV and 2×FYVE-labeled endosomes in parental HT1080 cells than in VPS29 KO cells (Fig. 6C and Fig. S5), mirroring the opposing effects of VPS KO on HCoV and IAV infection.

We hypothesized that such effect might be due to VPS29-dependent trafficking of antiviral proteins with activity against IAV to endosomes, such as IFITM3. We observed that IFITM3 knockdown enhanced IAV infection of parental HT1080 cells (Fig. S6A), in agreement with previous reports (39). However, IFITM3 knockdown augmented IAV infection in VPS29 KO cells (Fig. S6A), suggesting that the enhancement of IAV infection in VPS29 KO cells was not the result of loss of IFITM3 activity. Concordantly, IFITM3 localized to 2×FYVE-labeled endosomes in both WT and VPS29 KO cells, and there was no clear difference in localization (Fig. S6B). Overall, these findings suggest that enhanced IAV infection in VPS29 KO cells is due to increased egress from endosomes but is not due to altered localization and/or impaired activity of IFITM3.

10.1128/mBio.03002-21.6 FIG S6 The enhancement of Influenza infection by VPS29 KO is not mediated by loss of IFITM3 activity. (A) WT and VPS29 KO HT1080 cells were transfected with a pool of four control siRNAs (siCTL) or a pool of four siRNAs targeting IFITM3 (siIFITM3). Three days posttransfection, cells were infected with IAV. At 24 h postinfection, cells were stained, and the percentage of infected cells was determined by flow cytometry. (B) Representative images of WT and VPS29 KO HT1080 cells stably expressing V5-tagged IFITM3 and labeled with 2×FYVE-mSCAR. Download FIG S6, PDF file, 0.3 MB. Copyright © 2022 Poston et al. 2022 Poston et al. https://creativecommons.org/licenses/by/4.0/ This content is distributed under the terms of the Creative Commons Attribution 4.0 International license.

### Impairment of CoV and ebolavirus infection in VPS29 KO cells due to loss of endosomal cathepsin activity.

The aforementioned findings indicate that the reduced susceptibility to HCoV infection in VPS29 KO cells is spike specific and is the consequence of failed egress from endosomes. We hypothesized that this effect could be due to impaired spike processing by endosomal proteases during entry. We used HIV-1-based pseudotyped viruses to test the susceptibility of various CoV spikes to VPS29 KO and cathepsin inhibition using the drug E64d. As rVSV/SARS-CoV-2 bears a point mutation, R683G, which ablates the polybasic furin cleavage site, we tested pseudotypes bearing WT or R683G mutant spike proteins, as well as spike proteins from SARS-CoV and SARS-like CoV from bats and pangolins, which also do not contain polybasic cleavage sites (40).

**FIG 7:**
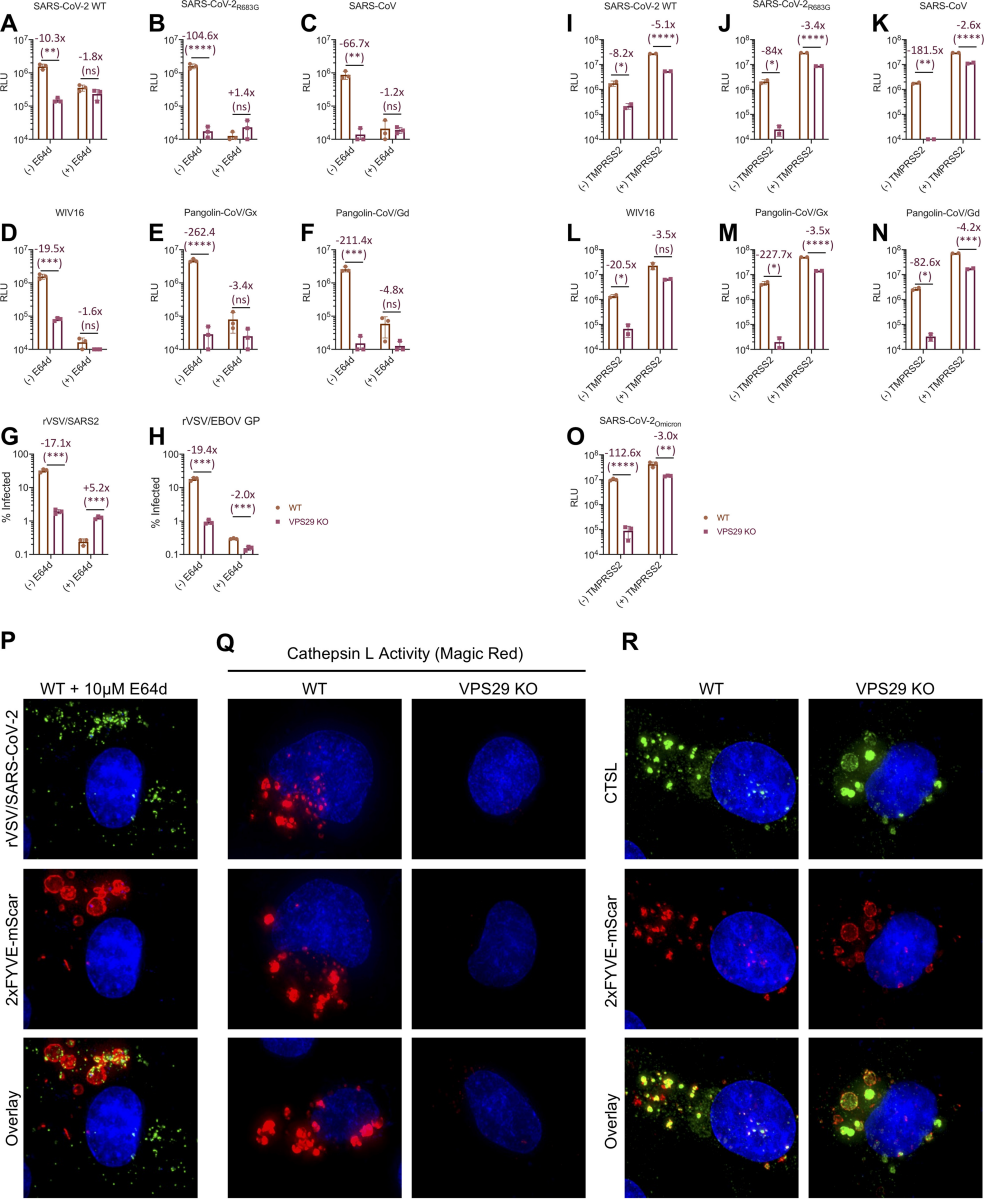
Impairment of CoV infection by VPS29 KO is influenced by the presence of a polybasic cleavage site and correlates with cathepsin inhibition. (A to F): WT and VPS29 KO HT1080 cells were treated with the indicated concentrations of E64d for 30 min before infection with HIV-1-based nanoluciferase reporter viruses pseudotyped with spike protein of WT SARS-CoV-2 (A), SARS-CoV-2_R683G_ (B), SARS-CoV (C), WIV16 (D), Pangolin-CoV/Gx (E), and Pangolin-CoV/Gd (F). At 48 hours postinfection (HPI), cells were harvested, and nanoluciferase activity was measured. The limit of detection of the HIV-1-based pseudoassay is 10^4^ relative light units (RLU). (G to H) WT and VPS29 KO HT1080 cells were treated with the indicated concentrations of E64d for 30 min before infection with rVSV/SARS-CoV-2 (G) or rVSV/EBOV GP (H). At 16 h postinfection, infected cells were enumerated by determined by flow cytometry. The limit of detection of the flow cytometry assay is 0.1% infection. Mean (bar graph) of three replicates (dots). Error bars indicate SD. Data shown are representative of at least two independent experiments. (I to O) WT and VPS29 KO HT1080 cells stably transduced to express TMPRSS2 were infected with HIV-1-based nanoluciferase reporter viruses pseudotyped with spike protein of WT SARS-CoV-2 (I), SARS-CoV-2_R683G_ (J), SARS-CoV (K), bat CoV/WIV16 (L), Pangolin-CoV/Gx (M), Pangolin-CoV/Gd (N), and SARS-CoV-2_Omicron_ (O). At 48 hpi, cells were harvested, and nanoluciferase activity was measured. Limit of detection of the HIV-1-based pseudoassay is 10^4^ RLU. Mean (bar graph) of two replicates (dots). Error bars indicate SD. Data shown are representative of at least two independent experiments. (P) Representative images of rVSV/SARS-CoV-2_NG-P_ infection in E64d-treated WT HT1080. 2×FYVE-mSCAR-labeled cells were treated with E64d for 30 min and then infected with rVSV/SARS-CoV-2_NG-P_ for 60 min. (Q) Representative images of WT and VPS29 KO HT1080 cells following 60-minute incubation with Magic Red cathepsin L activity kit. (R) Representative images of WT and VPS29 KO HT1080 cells stably expressing V5-tagged CTSL and labeled with 2×FYVE-mSCAR. Statistical test used is Student's *t* test between WT and KO cells. *, *P < *0.05; **, *P < *0.01; ***, *P < *0.001; ****, *P < *0.0001.

Pseudotypes bearing either the WT or the R683G mutant SARS-CoV-2 spike proteins were sensitive to VPS29 KO and cathepsin inhibition. However, cathepsin inhibition did not further decrease infection of VPS29 KO cells (Fig. 7A). The SARS-CoV-2_R683G_ (Fig. 7B), SARS-CoV (Fig. 7C), and the SARS-like bat (Fig. 7D) and pangolin viruses (Fig. 7E and F) that lack furin cleavage sites were more impacted by VPS29 KO and cathepsin inhibition than WT SARS-CoV-2. Indeed, in several instances, VPS29 KO and/or cathepsin inhibition resulted in undetectable infection by SARS-CoV-2_R683G_, SARS-CoV, and the SARS-like bat/pangolin CoVs. Similarly, infectivity assays utilizing rVSV/SARS-CoV-2 also revealed diminished infectivity upon cathepsin inhibition in parental HT1080, but no impairment of infection upon cathepsin inhibition in VPS29 KO cells (Fig. 7G).

That there was no further effect of cathepsin inhibition on CoV infection in VPS29 KO cells suggests that the effect of these two manipulations converge on a common pathway in promoting egress from endosomes. We thus hypothesized that VPS29 KO impedes CoV infection by impairing proper processing of spike by cathepsins. If this were indeed the case, then VPS29 KO should impair infection mediated by ebolavirus (EBOV) glycoprotein (GP), which is known to require processing by endosomal cathepsins (41). To test this, we performed infectivity assays in WT and VPS29 KO cells using a recombinant VSV expressing EBOV GP in lieu of VSV-G (rVSV/EBOV-GP) (42). Indeed, we observed a strong inhibition of rVSV/EBOV-GP infection upon either cathepsin inhibition or loss of VPS29 (Fig. 7H). This result suggests that the susceptibility of VPS29 KO is mediated by impaired cathepsin activity.

In order to further test if the primary effect of VPS29 KO on CoV infection was through impaired endocytic entry as a result of impaired cathepsin activity, we assessed the impact of VPS29 KO in cells overexpressing TMPRSS2. In cells expressing TMPRSS2, spike proteins can be cleaved and the fusion protein liberated at the cell surface, reducing dependence on endosomal cathepsins. Consistent with the hypothesis that VPS29 impairs endocytic viral entry, there was a dramatic reduction in the effect of VPS29 KO for SARS-CoV-2_R683G_, WIV16, Pangolin-CoV/Gx, and Pangolin-CoV/Gd and a more modest reduction for SARS-CoV-2_WT_ when target cells overexpressed TMPRSS2 (Fig. 7I to N). Importantly, since recent evidence indicates the Omicron variant of SARS-CoV-2 preferentially enters cells via the endocytic route rather than the TMPRSS2-dependent cell surface route (43, 44), we also included pseudotypes bearing the Omicron spike protein. Indeed, we observed robust inhibition of SARS-CoV-2_Omicron_ in VPS29 KO cells, 14× greater than that observed for WT spike (Fig. 7O). Additionally, the increase in infectivity observed in TMPRSS2-positive (TMPRSS2^+^) cells was less evident for Omicron than it was for the ancestral SARS-CoV-2_WT_ or SARS-CoV2_R683G_ (4.2-fold versus 15.5-fold or 14.3-fold, respectively). These findings suggest that the Omicron variant is particularly adapted to cellular entry through a VPS29-dependent endocytic route.

Consistent with the above conclusion, when parental HT1080 cells were treated with the cathepsin inhibitor E64d, infected with rVSV/SARS-CoV-2_NG-P_, and examined microscopically, we observed a phenotype similar to that seen in VPS29 KO cells (see Fig. 6A). Specifically, substantially more rVSV/SARS-CoV-2_NG-P_ punctae were evident within endosomes, and the endosomes appear enlarged with a similar appearance and morphology to those observed in VPS29 KO cells (Fig. 7P and Fig. S7).

10.1128/mBio.03002-21.7 FIG S7 Additional representative images demonstrating cathepsin inhibition results in rVSV/SARS2 remaining trapped in vesicles and that loss of other members of the retromer/CCC complex results in reduced cathepsin activity. (A) Additional representative images from Fig. 7P. 2×FYVE-mSCAR-labeled cells were treated with E64d for 30 minutes and then infected with rVSV/SARS-CoV-2_NG-P_ for 60 minutes. (B and C) Images of cells following 60-minute incubation with Magic Red cathepsin L activity kit. (B) HT1080 transfected with siRNA-targeting retromer proteins VPS29, VPS26A, and VPS35. (C) HT1080 WT or KO for the indicated gene. Download FIG S7, PDF file, 2.7 MB. Copyright © 2022 Poston et al. 2022 Poston et al. https://creativecommons.org/licenses/by/4.0/ This content is distributed under the terms of the Creative Commons Attribution 4.0 International license.

To directly test whether VPS29 KO results in impaired endosomal cathepsin activity, we measured endosomal cathepsin activity in WT and VPS29 KO HT1080 cells using a substrate that generates a fluorescent signal upon cleavage by cathepsin L (CTSL). Indeed, in WT cells, we observed a strong red fluorescence signal in vesicular structures, indicating high levels of cathepsin activity. However, in VPS29 KO cells, the red fluorescence signal was nearly absent, indicating impaired cathepsin activity in VPS29 KO cells (Fig. 7Q). A similar phenotype is seen in cells depleted of VPS26A and VPS35 by siRNA transfection or in which the CCC complex components were knocked out (Fig. S7B and C). To determine whether the loss of cathepsin L activity was the result of failed trafficking of cathepsins to the endolysosomal system, we performed immunofluorescence studies utilizing tagged cathepsin L in cells with endosomes labeled with 2×FYVE-mSCAR. Cathepsin L was clearly present in 2×FYVE-mSCAR-positive endosomes in VPS29 KO cells (Fig. 7R). Thus, these data suggest that the loss of cathepsin activity in VPS29 KO cells does not result from the absence of cathepsin in endosomes but, rather, altered endosomal conditions in VPS29 KO cells, such as increased pH, which reduces cathepsin activity therein.

## DISCUSSION

While the advent of robust, high-throughput screening modalities has generated a wealth of information regarding host-viral interactions, the underlying mechanism of action for many host proteins implicated by these screens remains incompletely understood. Here, using HCoV-OC43 as a model HCoV, we employed a genome-wide loss-of-function CRISPR screen to identify and characterize factors required for efficient CoV infection. In particular, we show that the retromer subunit protein VPS29 is required for productive infection by diverse CoVs in a variety of cell types. Other genome-wide screens using SARS-CoV-2 have also suggested a role for VPS29 and the CCC, as well as RAB7A, which recruits retromer to endosomes (26, 27, 45, 46) in HCoV infection.

While previous studies have hypothesized that the mechanism whereby VPS29/CCC complex facilitates SARS-CoV-2 infection is by maintaining cell surface expression of viral receptors (26, 27), our findings indicate a different role. Indeed, infection by HCoVs that use three distinct receptors was inhibited by VPS29/CCC KO, while there was no VPS29/CCC requirement for infection by IAV, adenovirus, or RSV, which should otherwise also be dependent on cell surface expression of their respective receptors. Indeed, HCoV-OC43’s purported receptor (9-*O*-acetylated sialic acid) is nearly identical to IAV’s receptor (sialic acid). Yet, while HCoV-OC43 infection is highly dependent on VPS29/CCC, IAV infection is either unaffected by these factors (in A549 cells) or hindered by them (in HT1080 and 293T cells).

A possible mechanism that might account for the enhancing effect of VPS29 deficiency on IAV infection could be retromer-dependent trafficking of endosomal cargo that antagonizes IAV infection. While this notion is consistent with our finding that showing incoming IAV accumulated in endosomes to a greater extent in normal than in VPS29 KO cells, the proteins that might be responsible for mediating this effect are unknown. Other possibilities include direct influence of retromer on IAV-containing endosomes. In this regard, retromer-dependent movement of human papillomavirus (HPV) to the TGN has been reported to involve direct interaction between retromer and the HPV L2 protein (47). While loss of retromer/WASH complex facilitated IAV infection in HT1080 cells, loss of other retromer-interacting proteins, such as SNX6 and Rab38, impaired IAV infection. Thus, distinct effector functions of VPS29 may have different infection-enhancing and inhibiting properties, with the overall effect depending on viral and/or cell type-specific characteristics. Differences in expression and/or activity of various VPS29 effector proteins may explain why VPS29 KO facilitates IAV infection in HT1080 and 293T cells, but not in A549 cells.

However, VPS29 KO impaired CoV infection in all cells tested, including primary lung cells. Nevertheless, we did observe some virus and cell type-specific differences. Specifically, loss of both retromer and retriever impaired HCoV-OC43 infection, while loss of retriever did not impair HCoV-NL63 or HCoV-229E or rVSV/SARS-CoV-2 infection. These findings suggest that there may be multiple roles for VPS29 in HCoV infection, with some CoVs requiring the effector functions of distinct VPS29-containing complexes. The precise requirement for distinct VPS29 functions could vary with cell type; for example, there was a decreased requirement for the CCC complex in HT1080 and 293T cells. Our finding that SARS-CoV-2_R683G,_ SARS-CoV, and bat/pangolin CoVs were all heavily impacted by both VPS29 KO and cathepsin inhibition suggests that these viruses are especially sensitive to endocytic function, in line with recent work demonstrating that mutation of the SARS-CoV-2 polybasic cleavage site drives virions to enter via the endocytic route (48). Additionally, our finding that the Omicron variant of SARS-CoV-2 is more heavily dependent on VPS29 than SARS-CoV-2_WT_ is in agreement with recent reports that the Omicron variant preferentially enters cells via the endocytic route (43, 44).

Based on our findings, it appears that a key feature of VPS29/retromer KO cells is elevation of the pH of endolysosomal compartments. This change should impair activation of cathepsins (49), thus impeding endosomal CoV spike as well as EBOV GP processing and egress from endosomes to initiate productive infection, consistent with our observations of incoming virions. In agreement with this model, others have shown that VPS35 deficiency results in reduced endosomal cathepsin activity (50). Here, however, we show that this reduced endosomal activity is likely due to perturbed endolysosomal pH rather than an absence of cathepsin zymogen trafficking to endosomes in VPS29 KO cells. We also speculate that VPS29 deficiency underlies increased cellular susceptibility to IAV infection by reducing virion exposure to destructive lysosomal proteases that may be either absent from the endosome due to impaired trafficking or rendered inactive by the elevated pH in these cells.

Importantly, our findings suggest that the exploration of cathepsin inhibitors, or other endosomal perturbing agents, is a promising target for novel drugs against CoVs, which remain a potentially serious emergent public health threat. Indeed, our finding that the Omicron variant is particularly sensitive to loss of VPS29 suggests that modulating this pathway pharmacologically could be an attractive candidate for new therapeutic strategies targeting this and future variants with similar properties.

## MATERIALS AND METHODS

### Cell culture.

HEK-293T (Homo sapiens; sex, female), A549 (H. sapiens; sex, male), HT1080 (H. sapiens, sex, male), MDCK (Canis familiaris), and Vero cells (Cercopithecus aethiops) were obtained from ATCC, and Huh7.5 cells (generously provided by Charles M. Rice) were maintained at 37°C and 5% CO_2_ in Dulbecco’s modified Eagle medium (DMEM; Gibco) supplemented with 10% fetal bovine serum. NHBE cells (H. sapiens) were obtained from ATCC (catalog no. ATCC PCS-300-010) and maintained at 37°C and 5% CO_2_ in airway epithelial cell basal medium (catalog no. ATCC PCS-300-030) supplemented with bronchial epithelial cell growth kit (catalog no. ATCC PCS-300-040). All cells were assessed for *Mycoplasma* contamination.

### Production of viral stocks.

HCoV-OC43 (strain, ATCC VR-759) and HCoV-229E (strain, ATCC VR-740) were obtained from Zeptometrix Corporation, and HCoV-NL63 (strain, Amsterdam I) was obtained from the Biodefense and Emerging Infections Research Resources Repository. Viral stocks were generated by propagation on Huh7.5 cells. The IAV strains A/WSN/33 (H1N1), A/Netherlands/602/2009 (H1N1)pdm09 (H1N1_2009 Netherlands_), and A/California/04/2009 (H1N1)pdm09 (H1N1_2009 California_) were propagated in MDCK cells in the presence of 3μg/mL TPCK-trypsin (Sigma). RSV strain A2-line19F expressing the red fluorescent protein monomeric Katushka 2 (mKate2) (51) was propagated in Vero cells. Adenovirus 5 was purchased from ATCC (VR-1516) and propagated in A549 cells. VSV_IND_eGFP was propagated on 293T cells (52). The replication-competent chimeric recombinant vesicular stomatitis virus encoding SARS-CoV-2 S and enhanced green fluorescent protein (eGFP), rVSV/SARS-2/GFP_2E1_, has been described previously (53) and was propagated on 293T-ACE2 cells. rVSV/EBOV-GP was propagated on Vero cells as previously described (42).

### CRISPR-Cas9 screening.

The human genome-wide Brunello library (15) in lentiCRISPRv2 was obtained from Addgene (catalog no. 73179) and amplified according to the depositor’s instructions. The resulting plasmid DNA was validated via next-generation sequencing (NGS) to confirm appropriate coverage and representation (the resulting library contained 0.0% undetected guides, and a skew ratio of the top 10% represented guides to the bottom 10% represented guides was 3.94, well below the recommended cutoff of 10 for an “ideal” library [54]). To generate lentiviral preparations of the Brunello library, 293T cells (6 × 10^6^ cells per 10-cm dish) were transfected with 6 μg lentiCRISPRv2-Brunello, 6 μg NL-gagpol, and 1.2 μg VSV-G using polyethyleneimine (PEI). Forty-eight hours posttransfection, supernatants were pooled and concentrated using Amicon Ultra centrifugal filters. Concentrated lentiviral preps were stored at −80°C and titrated on A549 cells based on puromycin resistance. Briefly, 10-fold serial dilutions (from 10^−1^ to 10^−6^) were used to transduce 40,000 A549 cells in a 24-well plate format. Forty-eight hours postransduction, cells were trypsinized and moved up to 6-well plates in the presence of 1.25 μg/mL puromycin. Nine days postransduction, cells were fixed and stained with crystal violet, and stained foci were counted to measure the number of cells surviving selection (i.e., those that were transduced with lentiCRISPRv2 harboring a puromycin resistance cassette). To perform the screen, 1.3 × 10^8^ A549 cells were transduced with lentiCRISPRv2-Brunello at an MOI of 0.3 in order to generate a population of single KO cells at high (>500×) coverage. Two days postransduction, cells were placed in selection with 1.25 μg/mL puromycin and passaged for 7 days until there were no untransduced cells remaining. Thereafter, in triplicates with 8 × 10^6^ cells per flask, A549-Brunello cells were infected or not with HCoV-OC43 at an MOI of 0.1 and passaged for 7 days until >95% infection-induced cell death occurred. Cellular gDNA was isolated using Zymogen Quick-DNA Midiprep Plus kit (Zymo Research) and sequencing libraries prepared via amplification of sgRNA loci utilizing F primers containing P5 and read 1 sequencing primer and an R primer containing P7, a barcode, and the multiplexing Index Read sequencing primer, as described in Joung et al. (54). The resulting libraries were gel purified, pooled, and sequenced on the Illumina HiSeq at Genewiz using 80 cycles of read 1 (forward) and 8 cycles of index 1 using standard Illumina sequencing primers.

### Pathway analysis of screen hits.

All 34 candidate genes were searched using the STRING database (https://string-db.org/) for functional enrichment of protein-protein interactions using default settings, except the minimum required interaction score was changed from medium confidence (0.400) to high confidence (0.700). Subsequently, genes were annotated with UniProt keywords (https://uniprot.org).

### Validation of CRISPR hits.

Individual sgRNAs targeting hits of interest were cloned into lentiCRISPRv2 via linearization with BsmBI followed by ligation of annealed oligonucleotides with compatible sticky ends using the primers VPS29 forward, caccgGGACATCAAGTTATTCCATG; VPS29 reverse, aaacCATGGAATAACTTGATGTCCc; CCDC22 forward, caccgCCGCAGGGTTGATCACACGC; CCDC22 reverse, aaacGCGTGTGATCAACCCTGCGGc; CCDC93 forward, caccgTAGAATCCAAAGCTGATCCA; CCDC93 reverse, aaacTGGATCAGCTTTGGATTCTAc; COMMD3 forward, caccgCTTGAAACATATCGACCCAG; and COMMD3 reverse, aaacCTGGGTCGATATGTTTCAAGc. As a control, unmodified (“empty vector”) lentiCRISPRv2, which does not harbor an sgRNA cassette, was used. Lentiviral preparations were obtained by transfecting 1 × 10^6^ 293Ts with 1 μg lentiCRISPRv2, 1 μg NL-gagpol, and 0.2 μg VSV-G using PEI. Two days posttransfection, supernatants were collected, filtered, and used to transduce 5 × 10^4^ A549-ACE2, HT1080-ACE2, 293T-ACE2, or NHBE cells. Two days postransduction, cells were trypsinized, placed in selection with 1.25 μg/mL puromycin, and passaged until there were no remaining viable untransduced cells. CRISPR KO was verified by Sanger sequencing and Western blot analysis.

### Infectivity assays.

A total of 1 × 10^4^ cells per well were seeded on a 96-well plate in triplicate. The next day, cells were infected with each virus at an MOI of ∼0.3. For HCoV-OC43, HCoV-NL63, and HCoV-229E, infected plates were incubated at 34°C for 24 h. For IAV, RSV, and adenovirus, infected plates were incubated at 37°C for 24 h. For rVSV/SARS-CoV-2, infected plates were incubated at 37°C for 16 h. Cells were then fixed in 4% paraformaldehyde (PFA). For rVSV/SARS-CoV-2 and RSV, which encode eGFP and mKate2 reporter genes, respectively, the number of infected cells was measured directly by flow cytometry. Otherwise, cells were immunostained for viral antigens. Briefly, cells were blocked with 5.0% fetal bovine serum in phosphate-buffered saline (PBS) and permeabilized with 0.5% saponin before a 30-min incubation as follows: for HCoV-OC43, anti-coronavirus group antigen antibody, nucleoprotein of OC-43 (1:1,000; Sigma; catalog no. MAB9013); for HCoV-NL63, anti-coronavirus NL63 (1:1,000; Eurofins; catalog no. M.30.HCo.B2D4); for HCoV-22, anti-coronavirus 229E (1:1,000; Eurofins; catalog no. M.30.HCo.B1E7); for IAV, IAV NP antibody, fluorescein isothiocyanate (FITC) (1:50; Invitrogen; catalog no. MA1-7322); and for adenovirus, adenovirus hexon antibody, FITC (1:50; Invitrogen; catalog no. MA1-7329). For unconjugated primary antibodies (HCoV-OC43, HCoV-NL63, and HCoV-229E), a secondary antibody conjugate AF-488 goat anti-mouse IgG (H+L) (1:1,000; Thermo) was used before infected cells were enumerated via flow cytometry.

### siRNA screening of VPS29 interactors.

A list of well-known VPS29 interactors (Baños-Mateos, 2019) (10) (Fig. 1) was selected and used to construct a targeted siRNA library constructed of a pool of four different gene-specific siRNA sequences (ON TARGETplus SMARTpool siRNA; Dharmacon).

### siRNA transfection.

siRNAs were reverse-transfected with 5 × 10^3^ HT1080-ACE2 using RNAiMAX (Thermo Scientific) according to the manufacturer’s protocol. Two or three days posttransfection, cells were infected at an MOI of ∼0.3 and processed as above.

### Plasmid construction.

The lentiviral expression vector CSIN was derived from CSIB (55) by exchanging the blasticidin resistance cassette with neomycin. Briefly, primers Neo_CSIB_F, AAAAACACGATGATAATATGGCCACAACCAATTGAACAAGATGGATTGCACGCAGGTTCT, and Neo_CSIB_R, AGCTTGATATCAAGCTTGCATGCCTGCAGGTCAGAAGAACTCGTCAAGAAGGCGATAGAA, were used to amplify the neomycin resistance cassette and assemble into CSIB linearized with and BstXI and SbfI using NEBuilder HiFi DNA Assembly (NEB). The 2×FYVE-mSCAR endosome labeling construct was constructed by adding 2 FYVE domains to the N terminus of mScarlet. FYVE domains were PCR amplified from the Hrs protein using primers FYVE_1_F, ACAGACTGAGTCGCCCGGGGGGGATCCGGCCGAGAGGGCCGCCACCGAGAGCGATGCCATGTTTGC; FYVE_1_R, GGCAGCAAACATGGCATCGCTCTCGGATCCTCCTCCTCCCTCCGCTTTCCTGTTCAGCTG; FYVE_2_F, CAGCTGAACAGGAAAGCGGAGGGAGGAGGAGGATCCGAGAGCGATGCCATGTTTGCTGCC; and FYVE_2_R, TCACTGCCTCGCCCTTGCTCACCATGGATCCTCCTCCTCCCTCCGCTTTCCTGTTCAGCT. mScarlet was PCR amplified using primers mSCAR_F, AGCTGAACAGGAAAGCGGAGGGAGGAGGAGGATCCATGGTGAGCAAGGGCGAGGCAGTGA, and mSCAR_R, GGGGGAGGGAGAGGGGCGGATCAGGCCAGAGAGGCCCTACTTGTACAGCTCGTCCATGCC. The resulting fragments were assembled into CSIB linearized with SfiI using NEBuilder HiFi DNA Assembly (NEB).

To generate rVSV/SARS-CoV-2_NG-P_, the spike coding DNA sequence (CDS) was reverse transcribed and PCR amplified from rVSV/SARS-2/GFP_2E1_ using primers P-NG-2E1-S-MluI_F, MLUAGAGATCGATCTGTTTCCTTGACACGCGTATGTTTGTGTTCCTGGTGCTGCTGCCA, and P-NG-2E1-S-NotI_R, AACATGAAGAATCTGTTGTGCAGGGCGGCCGCCTTACAACAGGAGCCACAGGAA.

The resulting fragment was ligated into VSV NG-P (56) plasmid linearized with MluI and NotI using T4 ligase (NEB).

### Reconstitution experiments.

A VPS29 coding sequence containing silent mutations in the sgRNA targeting sequence was purchased from IDT and cloned into CSIN using NEBuilder HiFi DNA Assembly (NEB). The VPS29_I91D_ and VPS29_L152E_ derivates were obtained via PCR mutagenesis using primers I91D forward, GGTCACCAAGTAGATCCTTGGGGA; I91D reverse, TCCCCAAGGATCTACTTGGTGACC; L152E forward, CCATCATTTGTGGAGATGGATATCCAGGC; and L152E reverse, GCCTGGATATCCATCTCCACAAATGATGG. The resulting constructs, including an empty vector CSIN used as a control, were used to transduce single-cell clones obtained from bulk empty vector (EV) or VPS29 KO HT1080-ACE2 via limiting dilution. Infectivity assays on the resulting cell lines were performed as described above.

### HIV/NanoLuc CoV pseudotype assays.

To generate HIV/Nanoluc CoV-pseudotyped particles, 5 × 10^6^ 293T cells were plated in 10 mL growth medium in a 10-cm dish. The next day, 7.5 μg pHIV-1_NL4-3_ ΔEnv-NanoLuc and 2.5 μg indicated CoV spike plasmid were transfected using PEI. The medium was changed after 8 h of incubation. At 48 h posttransfection, the supernatant was harvested, passed through a 0.22-μm polyvinylidene fluoride syringe filter (Millipore; catalog no. SLGVR33RS), aliquoted, and stored at −80°C. To perform NanoLuc assays with the resulting HIV/Nanoluc CoV-pseudotyped particles, a total of 1 × 10^4^ HT1080-ACE2 WT or VPS29 KO cells per well were plated in triplicate in a 96-well plate. The next day, ∼1 × 10^3^ infectious units of HIV/NanoLuc CoV-pseudotyped particles were added to cells and incubated at 37°C for 48 h. Thereafter, cells were harvested for NanoLuc luciferase assays using the Nano-Glo luciferase assay system (Promega; catalog no. N1150).

### pHrodo dextran endocytosis assay.

Cells were plated in a Nunc Lab-Tek II chamber slide (Thermo) at 5 × 10^3^ cells per well. The next day, cells were transduced with 2×FYVE-mSCAR to label endosomes. Forty-eight hours postransduction, cells were treated with pHrodo Green dextran 10,000 molecular weight (MW) (Thermo; catalog no. P35368) at a concentration of 100 μg/mL for 60 min. Alternatively, unlabeled cells were treated with an equal ratio of pHrodo Red dextran 10,000 MW (Thermo; catalog no. P10361) and AF-488 dextran 10,000 MW (Thermo; catalog no. D22910). Thereafter, cells were washed 3× in PBS and placed in live cell imaging solution (Thermo; catalog no. A14291DJ). For 2×FYVE-labeled cells, images were acquired on a DeltaVision OMX SR imaging system using a ×60 widefield oil immersion objective (Olympus) with an exposure time of 50 ms, 5.0% transmission for the AF-488 channel, an exposure time of 50 ms, 10% transmission for the A568 channel, and an exposure time of 150 ms, 10% transmission for the DAPI (4′,6-diamidino-2-phenylindole) channel. For codextran-treated cells, images were acquired on a DeltaVision OMX SR imaging system using a ×60 widefield oil immersion objective (Olympus) with an exposure time of 25 ms, 10.0% transmission for the AF-488 channel, an exposure time of 50 ms, 10% transmission for the A568 channel, and an exposure time of 200 ms, 10% transmission for the DAPI channel.

### Microscopy of rVSV/SARS-CoV-2-infected cells.

Cells were plated in a Nunc Lab-Tek II chamber slide (Thermo) at 5 × 10^3^ cells per well. The next day, cells were transduced with 2×FYVE-mSCAR to label endosomes. For rVSV/SARS-CoV-2_NG-P,_ 48 h postransduction, cells were treated with 5 μM E64d (Sigma-Aldrich; catalog no. E8640-250UG) for 30 min, followed by inoculation with rVSV/SARS-CoV-2_NG-P_ at an MOI of 2. Sixty minutes postinfection, cells were washed 3× with PBS and fixed in 4% PFA. Alternatively, unlabeled cells were treated with pHrodo Red dextran and infected with rVSV/SARS-CoV-2_NG-P_ for 60 min. Sixty minutes postinfection, cells were washed 3× with PBS and imaged in live cell imaging solution. For cells with 2×FYVE-labeled endosomes, images were acquired on a DeltaVision OMX SR imaging system using a ×60 widefield oil immersion objective (Olympus) with an exposure time of 50 ms, 10% transmission for the AF-488 channel, an exposure time of 100 ms, 10% transmission for the A568 channel, and an exposure time of 150 ms, 10% transmission for the DAPI channel. For cells with dextran Red-labeled endosomes, images were acquired on a DeltaVision OMX SR imaging system using a ×60 widefield oil immersion objective (Olympus) with an exposure time of 50 ms, 10% transmission for the AF-488 channel, an exposure time of 50 ms, 10% transmission for the A568 channel, and an exposure time of 200 ms, 10% transmission for the DAPI channel.

### Influenza virus immunofluorescence.

Cells were plated in a Nunc Lab-Tek II chamber slide (Thermo) at 5 × 10^3^ cells per well. The next day, cells were transduced with a construct expressing 2×FYVE-mSCAR to label endosomes. Forty-eight hours postransduction, cells were infected with IAV at an MOI of ∼10. Sixty minutes postinfection, cells were washed with PBS, fixed in 4% PFA, permeabilized with 0.1% Triton X-100, blocked with fetal bovine serum (FBS), and stained for influenza virus nucleoprotein (1:200; Abcam; catalog no. ab128193) and antibody conjugate AF-488 goat anti-mouse IgG (H+L) (Thermo; 1:1,000). Images were acquired on a DeltaVision OMX SR imaging system using a ×60 widefield oil immersion objective (Olympus) with an exposure time of 50 ms, 5.0% transmission for the AF-488 channel, an exposure time of 100 ms, 10% transmission for the A568 channel, and an exposure time of 100 ms, 10% transmission for the DAPI channel.

### Quantification of fluorescence microscopy.

For each cell, regions of interest (ROIs) corresponding to labeled endosomes were defined using the freehand selection tool in Fiji. Quantification of mean fluorescence intensity inside each ROI was determined using the “measure” command. For punctae quantification, the number of punctae inside each ROI was counted and summed to give the total number of punctae inside ROIs for each cell. Additionally, the total number of punctae outside ROIs in each cell was measured. The reported percentage of virus in endosomes corresponds to

$$\frac{\text{number of punctae inside ROIs}}{\text{number of punctae inside ROIs }+\text{ number of punctae outside ROIs}}\times 100$$

### Cathepsin L activity assay.

Cells were plated 2 × 10^4^ cells per well in a Nunc Lab-Tek II chamber slide (Thermo). The next day, intracellular cathepsin L activity was detected using the Magic Red Cathepsin L assay kit (Bio-Rad; catalog no. ICT941). Briefly, cells were incubated in 1× Magic Red and Hoechst 33342 stain for 30 min and then washed 3× with PBS before being placed in live cell imaging solution (Thermo; catalog no. A14291DJ). Images were acquired on a DeltaVision OMX SR imaging system using a ×60 widefield oil immersion objective (Olympus) using an exposure time of 50 ms, 10% transmission for the A568 nm channel, and an exposure time of 100 ms, 10% transmission for the DAPI channel.

### Cathepsin L localization staining.

The coding sequence of CTSL was tagged with a 3′V5 and cloned into CSIN using NEBuilder HiFi DNA Assembly (NEB) with primers CTSL_3′_V5_F, ACAGACTGAGTCGCCCGGGGGGGATCCGGCCGAGAGGGCCGCCACCATGAATCCTACACTCATCCTTGC, and CTSL_3′_V5_R, GGGGGAGGGAGAGGGGCGGATCAGGCCAGAGAGGCCTCACGTAGAATCGAGACCGAGGAGAGGGTTAGGGATAGGCTTACCCACAGTGGGGTAGCTGGCT. Cells stably expressing this 3′V5-tagged CTSL were plated in a Nunc Lab-Tek II chamber slide (Thermo) at 5 × 10^3^ cells per well. The next day, cells were transduced with a construct expressing 2×FYVE-mSCAR to label endosomes. Forty-eight hours postransduction, cells were fixed in 4% PFA, permeabilized with 0.1% Triton X-100, blocked with FBS, and stained for V5 (Invitrogen; catalog no. 46-0705; 1:1,000) and antibody conjugate AF-488 goat anti-mouse IgG (H+L) (Thermo; catalog no. 1:1,000). Images were acquired on a DeltaVision OMX SR imaging system using a ×60 widefield oil immersion objective (Olympus) with an exposure time of 50 ms, 5.0% transmission for the AF-488 channel, an exposure time of 100 ms, 10% transmission for the A568 channel, and an exposure time of 100 ms, 10% transmission for the DAPI channel.

### Statistical analysis.

GraphPad Prism 9 software was used to carry out all statistical analyses.
