# Supplementary figures and images for: VPS29 Exerts Opposing Effects on Endocytic Viral Entry
Source: mBio. 2022 Mar 1;13(2):e03002-21. doi: 10.1128/mbio.03002-21 (PMC8941944; doi:10.1128/mbio.03002-21)

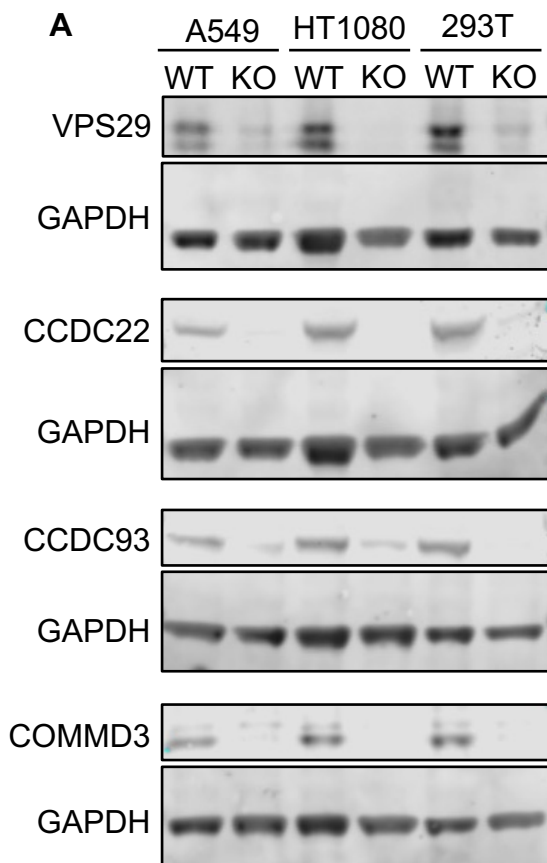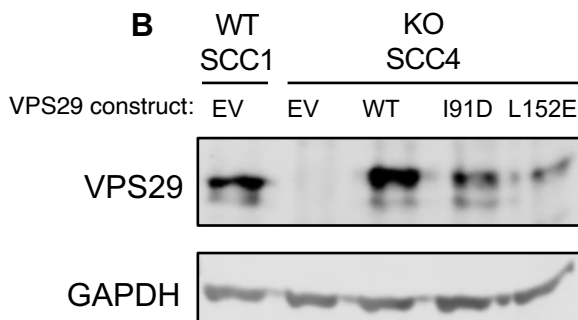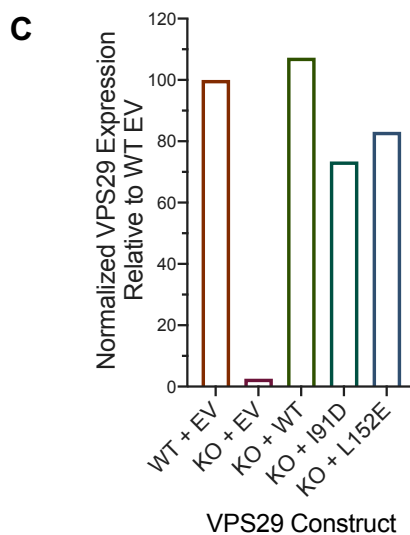

Supplement: FIG S1 [file mbio.03002-21-sf001.pdf]

**A**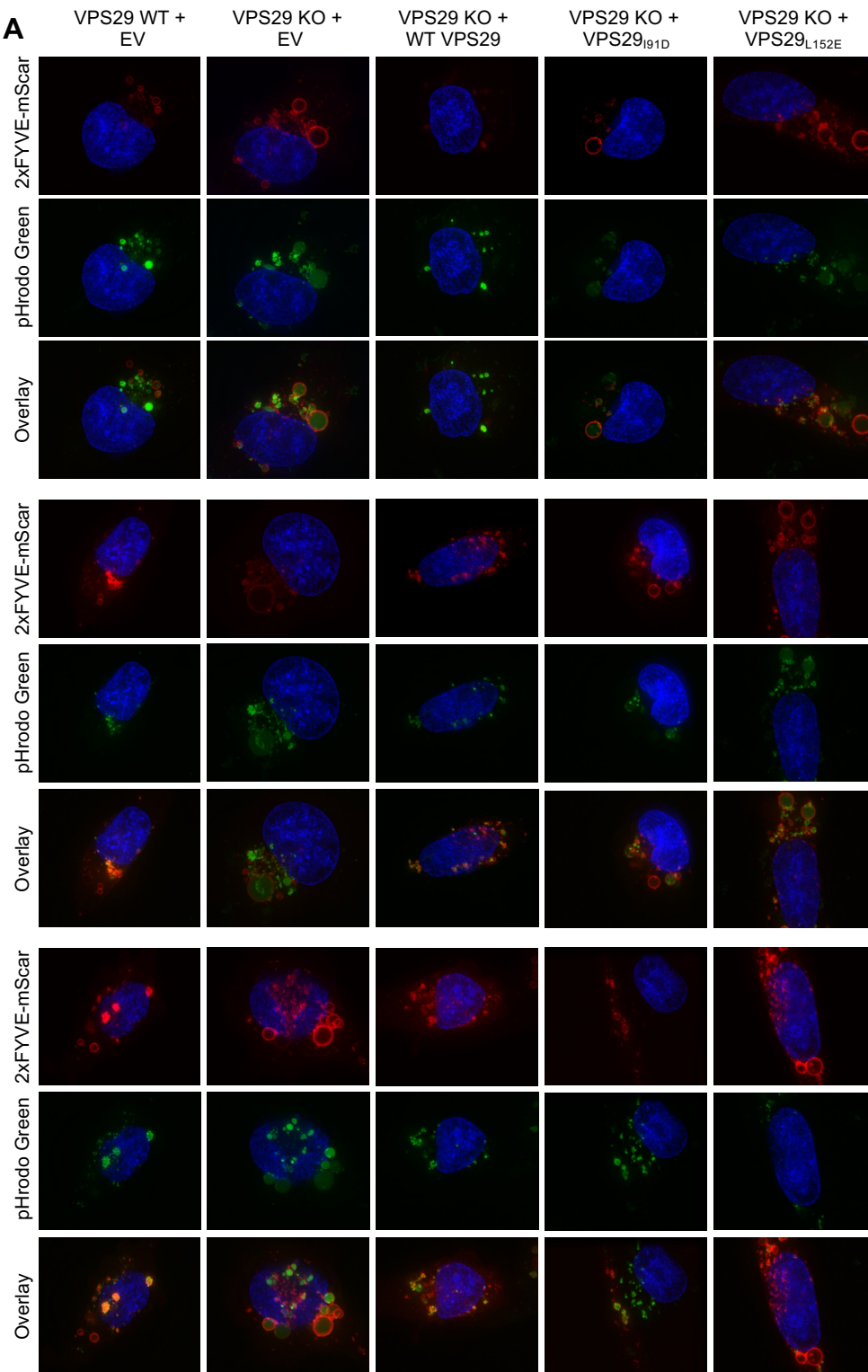**B**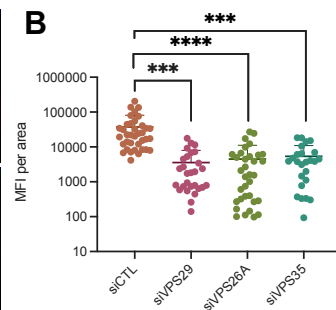**C**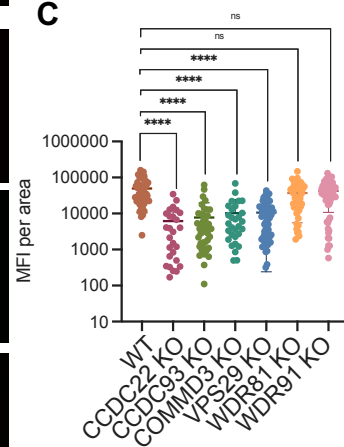

Supplement: FIG S2 [file mbio.03002-21-sf002.pdf]

Dextran AF-488  
(Non-pH dependent)

pHrodo Red Dextran  
(pH dependent)

Overlay

WT

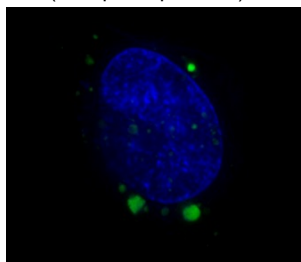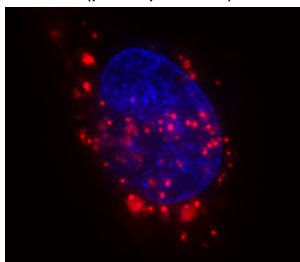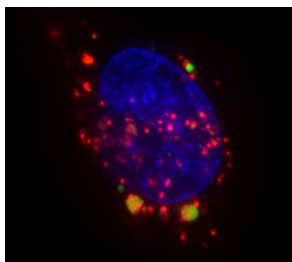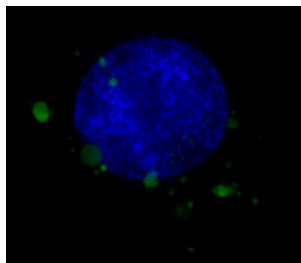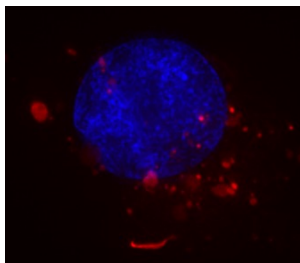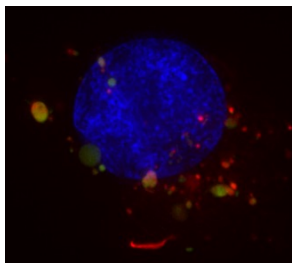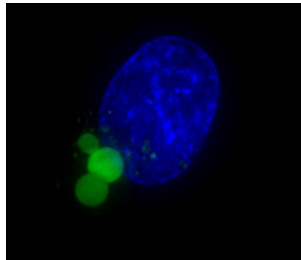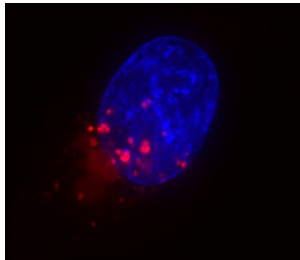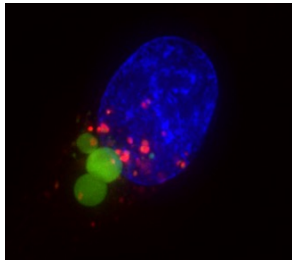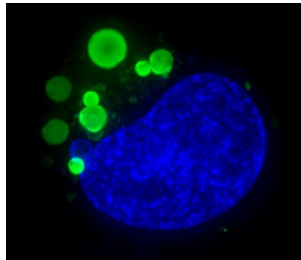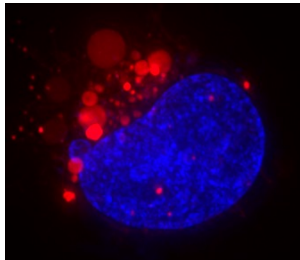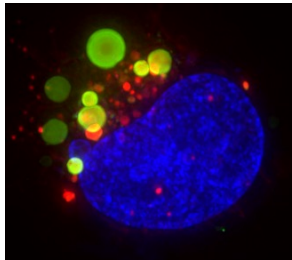

VPS29 KO

Supplement: FIG S3 [file mbio.03002-21-sf003.pdf]

**A**

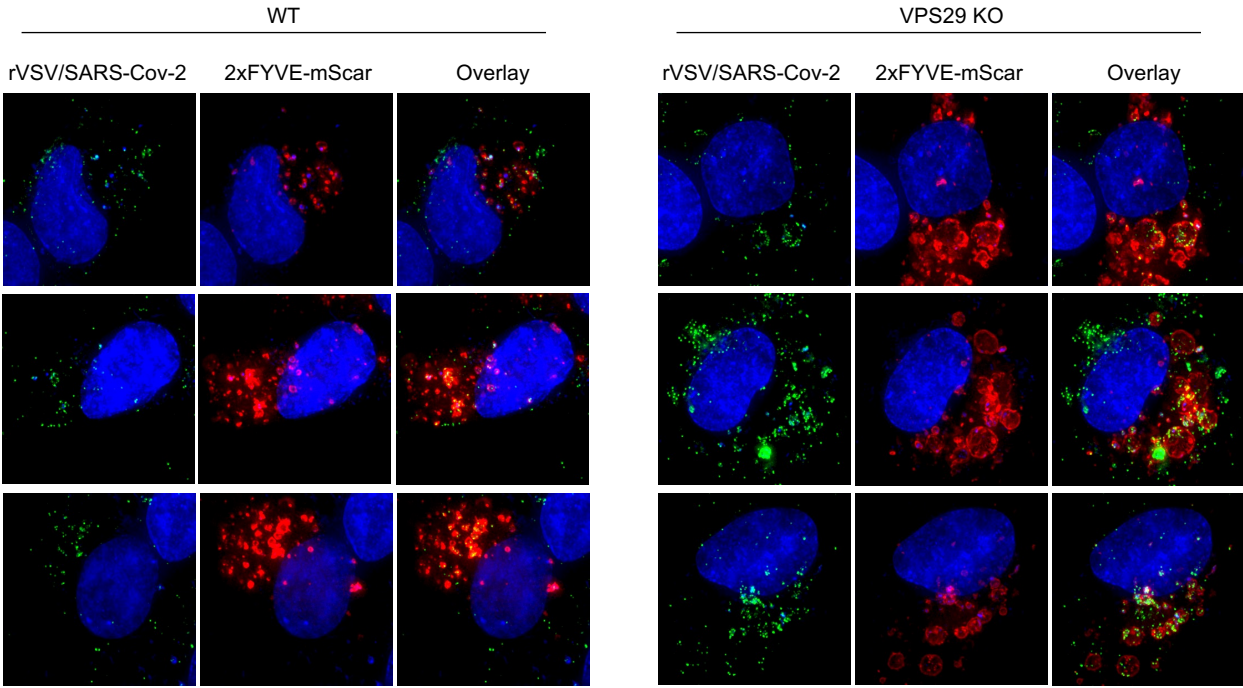

**B**

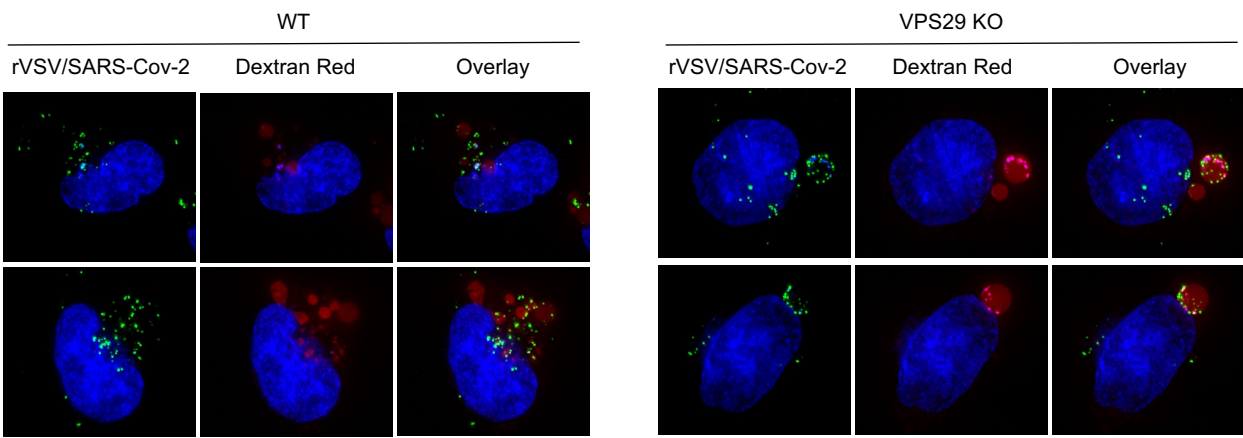

Supplement: FIG S4 [file mbio.03002-21-sf004.pdf]

WT

IAV

2xFYVE-mScar

Overlay

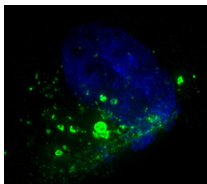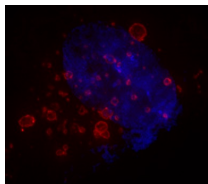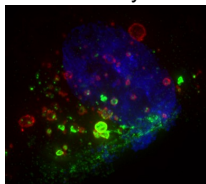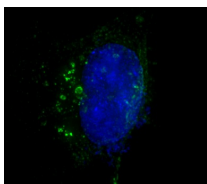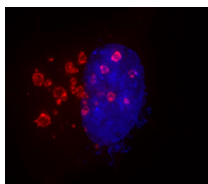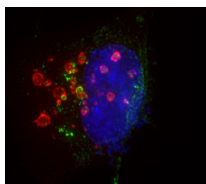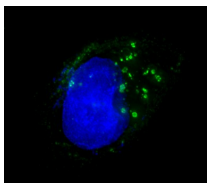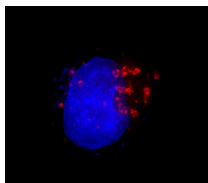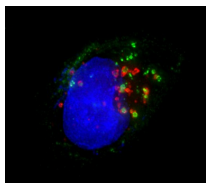

VPS29

IAV

2xFYVE-mScar

Overlay

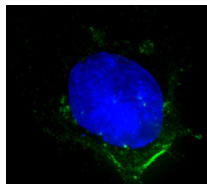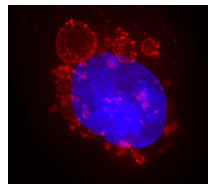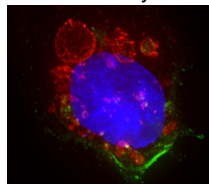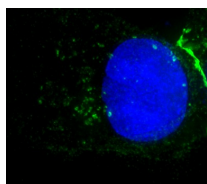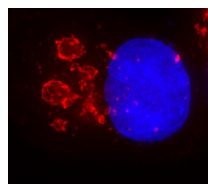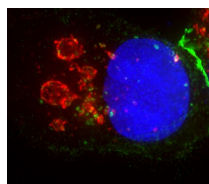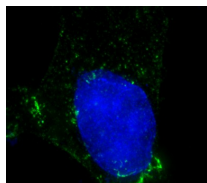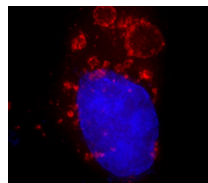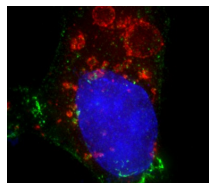

Supplement: FIG S5 [file mbio.03002-21-sf005.pdf]

**A**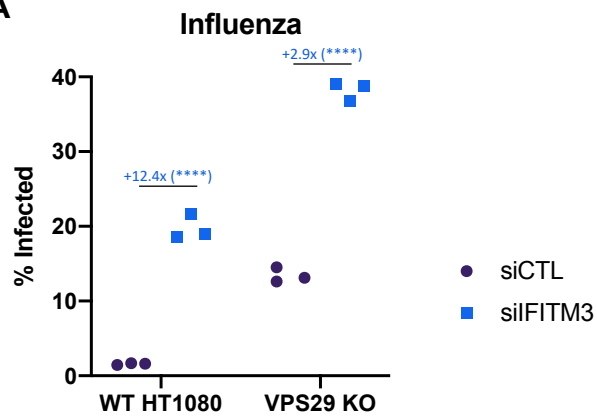**B**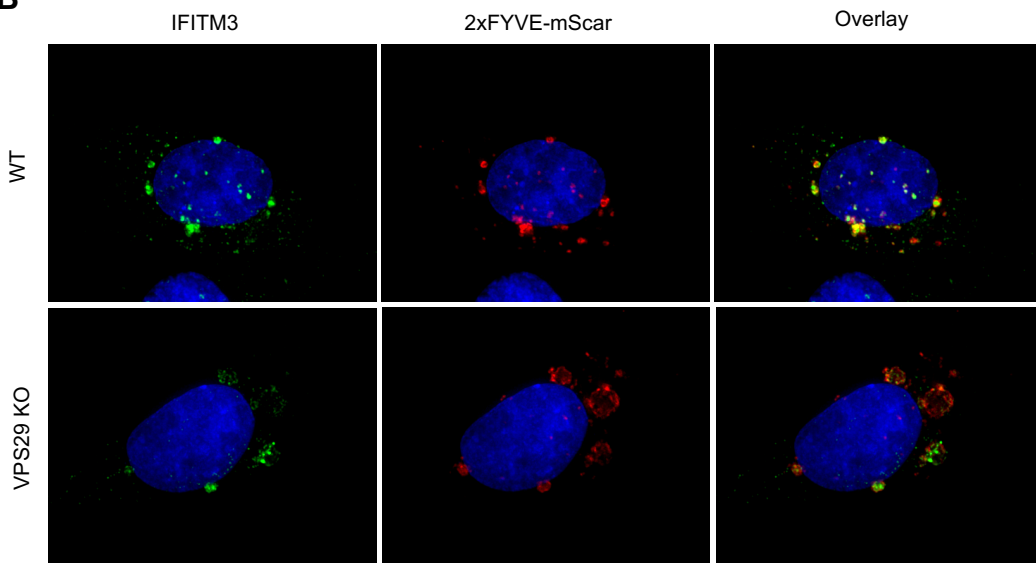

Supplement: FIG S6 [file mbio.03002-21-sf006.pdf]

**A** WT + 10μM E64D

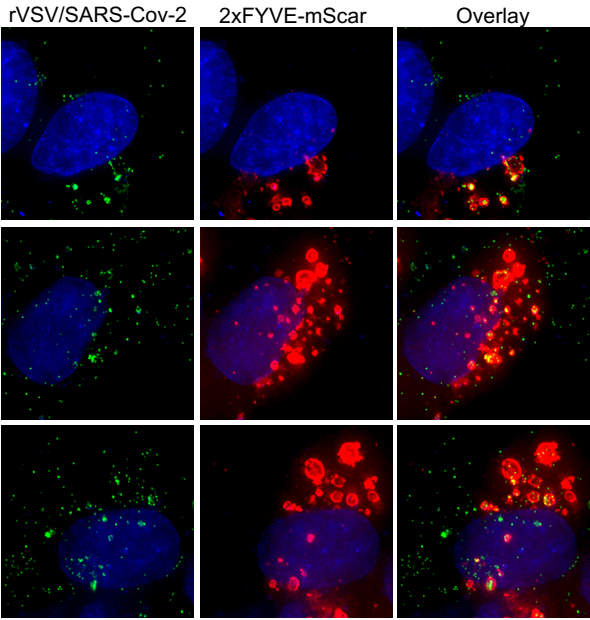

**B** Cathepsin L Activity (Magic Red)

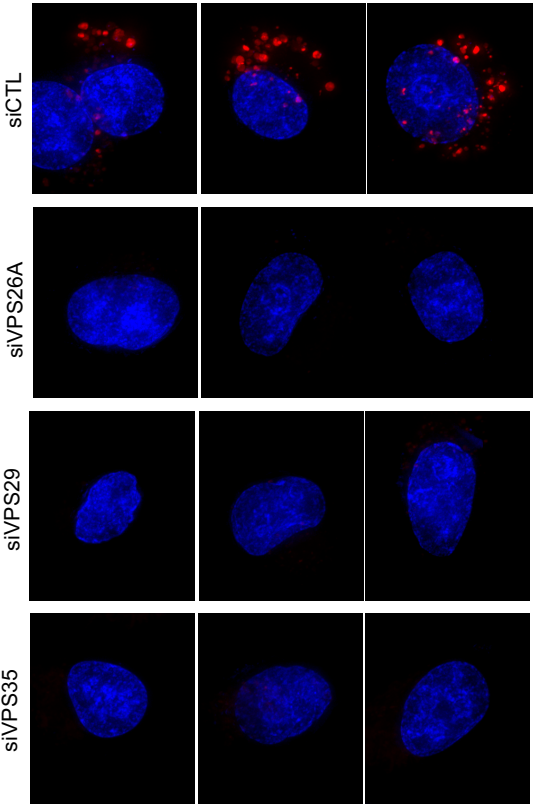

**C** Cathepsin L Activity (Magic Red)

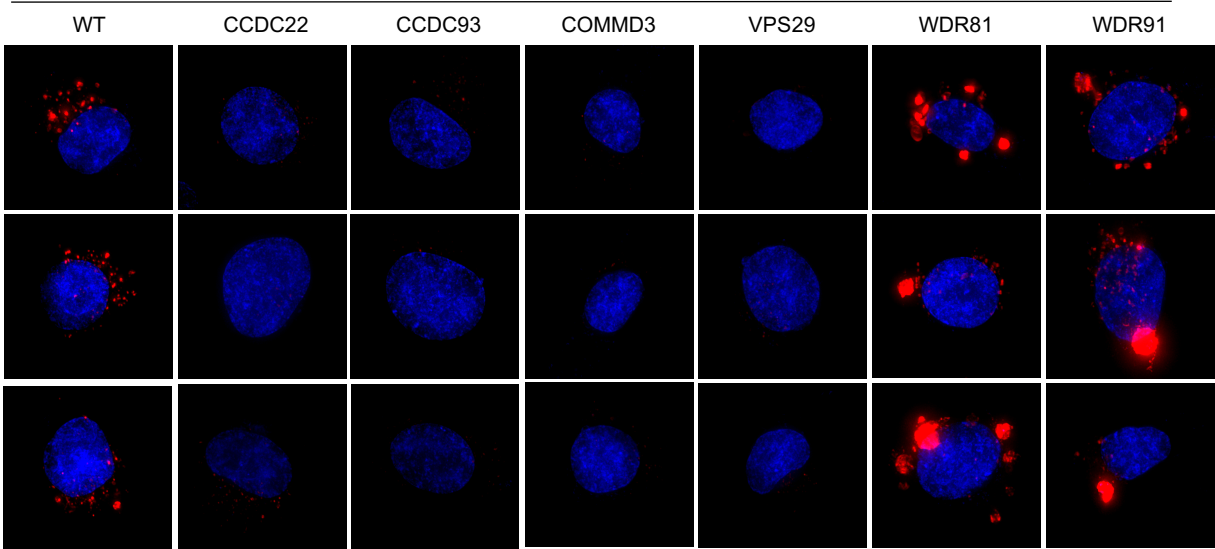

Supplement: FIG S7 [file mbio.03002-21-sf007.pdf]
